# Divergence of photosynthetic strategies amongst marine diatoms

**DOI:** 10.1371/journal.pone.0244252

**Published:** 2020-12-28

**Authors:** Nerissa L. Fisher, Douglas A. Campbell, David J. Hughes, Unnikrishnan Kuzhiumparambil, Kimberly H. Halsey, Peter J. Ralph, David J. Suggett

**Affiliations:** 1 Climate Change Cluster, University of Technology Sydney, Ultimo, New South Wales, Australia; 2 Department of Biology, Mount Allison University, Sackville, New Brunswick, Canada; 3 Department of Microbiology, Oregon State University, Corvallis, Oregon, United States of America; University of Nantes, FRANCE

## Abstract

Marine phytoplankton, and in particular diatoms, are responsible for almost half of all primary production on Earth. Diatom species thrive from polar to tropical waters and across light environments that are highly complex to relatively benign, and so have evolved highly divergent strategies for regulating light capture and utilization. It is increasingly well established that diatoms have achieved such successful ecosystem dominance by regulating excitation energy available for generating photosynthetic energy via highly flexible light harvesting strategies. However, how different light harvesting strategies and downstream pathways for oxygen production and consumption interact to balance excitation pressure remains unknown. We therefore examined the responses of three diatom taxa adapted to inherently different light climates (estuarine *Thalassioisira weissflogii*, coastal *Thalassiosira pseudonana* and oceanic *Thalassiosira oceanica*) during transient shifts from a moderate to high growth irradiance (85 to 1200 μmol photons m^-2^ s^-1^). Transient high light exposure caused *T*. *weissflogii* to rapidly downregulate PSII with substantial nonphotochemical quenching, protecting PSII from inactivation or damage, and obviating the need for induction of O_2_ consuming (light-dependent respiration, LDR) pathways. In contrast, *T*. *oceanica* retained high excitation pressure on PSII, but with little change in RCII photochemical turnover, thereby requiring moderate repair activity and greater reliance on LDR. *T*. *pseudonana* exhibited an intermediate response compared to the other two diatom species, exhibiting some downregulation and inactivation of PSII, but high repair of PSII and induction of reversible PSII nonphotochemical quenching, with some LDR. Together, these data demonstrate a range of strategies for balancing light harvesting and utilization across diatom species, which reflect their adaptation to sustain photosynthesis under environments with inherently different light regimes.

## Introduction

Diatoms account for the majority of marine primary production [1, 2] and are ubiquitous across aquatic environments [3], from tropical to polar regions, and from highly dynamic coastal and upwelling habitats to more stable oceanic waters. Adaptation of diatoms to these environments has resulted in their evolution of photosynthetic machinery optimized to very different light regimes caused by short-term (e.g. clouds, sun flecks, diel cycle) and long-term (e.g. seasonal) processes, as well as positioning relative to water column thermal and nutrient gradients [4, 5]. Whilst the overall success of diatoms appears driven by complex acclimation processes trending towards a light level that is close to an average irradiance within the mixed layer of a given water body [6, 7], routine exposure to stochastic high light episodes requires dynamic photoprotective capacity [8–11].

Diatoms use multiple processes to regulate photosynthesis, including modifying the ultrastructure (and thus the excitonic connectivity) of pigments and proteins, which together regulate the flow of excitation energy reaching the electron carrier system [8, 12]. Failure to regulate excess excitation energy either as photons reaching the reaction centre or as harvested energy (i.e. electrons) within the electron transport chain can increase the probability of photo-inactivation of photosystem II (PSII, [13]), likely from the generation of reactive oxygen species [14], ultimately leading to a decrease in net primary productivity and growth. It is therefore paramount that photoautotrophs have mechanisms to dissipate excess light energy and photo-protect the photosystems, and associated metabolism, while maintaining photosynthesis to support growth. One of the most important mechanisms for rapid (on the order of seconds to minutes) regulation of photochemistry under high light is non-photochemical quenching (see [15] review), parameterized herein as a yield, YNPQ (equivalent to ΦNPQ [16] and conceptually similar to [1-Q] [17]), whereby excitation energy in excess of the photosynthetic capacity, is safely dissipated as heat by light harvesting pigment complexes associated with PSII (LHCII, [18, 19]). YNPQ is a regulated process and contrasts with unregulated non-photochemical quenching, parameterized by YNO (equivalent to ΦNO, [16]). YNO comprises constitutive thermal losses [16] as well as intrinsic losses (*sensu* [20]).

As with all algae, light harvesting pigments in diatoms are connected to the PSII reaction centres (RCIIs) with an embedded oxygen evolving complex (OEC) that splits water to release electrons for use in photochemistry. Alterations in carotenoid pigment composition are regulated through the xanthophyll cycle (XC) and interact with particular photo-protective light harvesting complex protein isoforms (LHCXs) to lower the energetic transfer efficiency from antennae pigments to RCII [21–24]. When light is transiently in excess, induction of nonphotochemical quenching occurs whereby the accumulation of a proton gradient, and consequently ΔpH, across the thylakoid membrane drives the de-epoxidation of XC pigments, via two evolutionarily divergent protection pathways, diadinoxanthin (Dd, light harvesting) to diatoxanthin (Dt, photo-protective) [25] which dominates in diatoms and/or violaxanthin to zeaxanthin [26–28] which dominates in green lineages.

Exposure to high light triggers synthesis of LHCX isoforms that localize closely to the RCII core, resulting in core complex associated non-photochemical quenching [21, 24, 29]. Sustained high light exposure, in turn, drives accumulation of accessory pigments [24], which may also result from the continued accumulation of LHCXs [12, 23, 30] generating longer-lived, more slowly reversible, forms of nonphotochemical quenching. Nevertheless, diatoms typically maintain a constitutive capacity to build substantial but reversible nonphotochemical quenching through the XC [12]. Recent work using mutants of the pennate diatom *P*. *tricornutum* [31] showed that all capacity for rapidly reversible nonphotochemical quenching can be explained by LHCXs and the XC operating in concert and that, at least for *P*. *tricornutum*, longer-lived nonphotochemical quenching generally signifies the build-up of photo-inhibition that can only be reversed through turnover of PSII protein subunits [32]. An additional process—detachment of LHCXs from the RCIIs to invoke “super quenching”–appears to be a relatively minor process in *P*. *tricornutum*, suggesting that the energy trapping efficiency of the antenna does not out-compete that of the RCIIs [33, 34]. In other diatoms, however, antennae detachment appears to sustain nonphotochemical quenching [35–37], that in turn overlaps kinetically with photoinactivation and repair [13, 38].

Dynamic light regimes appear to select for phytoplankton taxa with different strategies of nonphotochemical quenching to optimize cell growth and survival, as demonstrated in a recent comparative assessment of various microalgal species and ecotypes [39]. Environments characterized by particularly large light fluctuations include shallow waters that are inhabited by both benthic diatoms and pelagic estuarine/coastal diatoms. Interestingly, the strategies used to deal with dynamic high light are quite different within niche-specific diatom groups, whereby non-motile benthic diatoms employ rapidly reversible nonphotochemical quenching through XC—presumably to cope with more variable light fields [8, 40]–whereas motile benthic diatoms preferentially employ slower, sustained non-photochemical quenching [21, 29]. This pattern has been further confirmed comparing Artic diatoms [41]. Within pelagic species, the coastal taxon *Skeletonema costatum* exhibits inherently less capacity for sustained non-photochemical quenching than the estuarine taxon *Phaeodactylum tricornutum*, but these alternate managements of excitation pressures are compensated by different capacities for PSII repair [42]. Here, maintaining a greater proportion of “active” PSIIs but lower capacity for non-photochemical quenching, presumably, places more pressure on electron carriers downstream of PSII to dissipate the transient accumulation of excessive excitation energy within the photosynthetic electron transport chain.

Microalgae exposed to supra-optimal light can further deal with excessive excitation energy through “alternative electron flows” downstream of PSII (e.g. [25, 43–45]). Numerous alternative electron pathways have been described, including electron cycles around PSII [46–48] and PSI [49] that do not consume O_2_, or alternative midstream terminal oxidase (MOX, [50]) pathways downstream of PSII (e.g. plastid terminal oxidase, PTOX, [51, 52]) or of PSI (Mehler-Ascorbate-Peroxidase, [53, 54]; Flavodiiron proteins, [55]) that consume electrons and O_2_. It has been suggested that such up-regulation of alternative electron flow directly feeds back to non-photochemical quenching generation at PSII by generating ΔpH—a key trigger of antennae-based non-photochemical quenching processes [52]. In spite of the potential importance of these electron pathways, relatively little is known as to whether they operate to sustain photo-protective capacity in diatoms.

Mehler Ascorbate Peroxidase activity (Mehler for brevity) is an alternative electron sink following PSI, consuming O_2_ to ultimately re-generate H_2_O (e.g. [54, 56]) and appears to be a significant route of total O_2_ uptake in the light amongst diatoms [57]. For example, 60% oxygen uptake via Mehler activity was observed for *Thalassiosira pseudonana* [58] and *Cylindrotheca* [59]. However, other reports have suggested a significant role for mitochondrial alternative oxidase (AOX; e.g. *Thalassiosira weissflogii*, [54]), which can, in turn, supply energy to chloroplast-protective processes [60]. Photorespiration related to RUBISCO oxidase function is often considered a negligible source of energy dissipation in diatoms as they have evolved carbon concentrating mechanisms [61, 62]. Importantly, Mehler, but not AOX, directly supports chloroplast proton motive force pathways that directly contribute to signalling photo-protection through the light harvesting apparatus (see [45]). At present, it remains unexplored whether and how the modulation of O_2_ consumption, as a means to balance excess excitation pressure, can be reconciled with differential capacities for non-photochemical protection amongst diatoms.

Here we initially examined allocation of excitation energy to non-photochemical vs. photochemical pathways across a broad panel of diatoms to uncover divergent strategies. We then analyzed three representative diatom taxa (*Thalassioisira weissflogii*, *Thalassiosira pseudonana*, *Thalassiosira oceanica*) from ecologically distinct light environments (estuarine, coastal, open ocean, respectively) to determine their balance of photo-protective strategies through XC versus PSII repair capacity, and whether species with higher capacities for non-photochemical quenching exhibited lower reliance on induction of light-dependent O_2_ consumption (light-dependent respiration, LDR). We therefore screened *T*. *weissflogii*, *T*. *pseudonana* and *T*. *oceanica* for (i) pigment content and de-epoxidation activity, (ii) PSII photo-inactivation and repair rate constants, and (iii) LDR upon transient exposure to high light, relative to the growth irradiance. Together these data demonstrate that diatom species from different ecological niches have highly divergent energy allocation strategies to cope with high light exposure.

## Materials and methods

### Culture conditions and growth

Seven diatoms, including *Phaeodactylum tricornutum* (CCMP 632), *Chaetoceros muelleri* (CCMP 1316), *Ditylum brightwellii* (CS 131), *Thalassiosira rotula* (CCMP 3264), *Thalassiosira pseudonana* (CCMP 1335), *Thalassiosira weissflogii* (CCMP 1336), and *Thalassisosira oceanica* (CCMP 1005), were initially screened for their response characteristics using chlorophyll fluorescence metrics. All species were grown as semi-batch cultures using f/2+Si medium [63] supplemented with Na_2_SeO_3_ at 0.17 μM concentration [57]. Nitrate and phosphate concentrations in the media reservoir were 250 and 50 μM, respectively [64]. Cultures were grown over 12:12 L:D cycle under 85 μmol photons m^-2^ s^-1^ at 20°C and maintained in exponential phase for 7–10 generations before sampling. Light was supplied by cool-white fluorescent tubes and intensities were measured using a LI-COR photometer (LI-250A, Nebraska, USA) equipped with a 4π spherical quantum sensor. Data were collected from independent biological replicates for cells maintained under exponential phase of growth.

### Photophysiology

Photophysiological assessment was performed using a FastOcean Fast Repetition Rate fluorometer (FRRf; S/N: 12-8679-007) fitted with a FastACT laboratory base unit (Chelsea Technologies Group Ltd, London, U.K), largely as per previous protocols [17, 65]. A protocol cumulatively applying 100 flashlets (1 μs flashlets with 2 μs intervals) was used to drive single turnover (ST) closure of RCIIs to generate a fluorescence induction curve. This induction was immediately followed by a relaxation phase of 40 flashlets (1 μs flash with 50 μs intervals). The biophysical model of Kolber *et al*. [66] was then fitted to the generated fluorescence transient to extract minimal (*F*_o_) and maximum (*F*_m_) fluorescence, the functional absorption cross section of PSII (σ_PSII_, nm^2^ PSII^-1^) and the lifetime for re-opening of PSII (τ, μs). All ST measurements were performed using blue excitation LED (450 nm) and a total of 40 induction/relaxation sequences were conducted per acquisition, with 150 ms intervals between sequences. Samples were taken from growth light cultures and shifted to low light (<10 μmol photons m^-2^ s^-1^) for 5 min to relax non-photochemical quenching processes before fluorescence measurements were made. Values of σ_PSII_ and maximum PSII photochemical efficiency (*F*_v_*/F*_m_, dimensionless) were then immediately measured on samples transferred to darkness, with *F*_v_*/F*_m_ calculated as:

$$\frac{F_{v}}{F_{m}}\;=\;\frac{F_{m}-F_{o}}{F_{m}}$$ (1)

Fluorescence-light response curves (FLCs) were performed in triplicate over 12 light steps of increasing irradiance ranging from 0–1304 μmol photons m^-2^ s^-1^, provided by a cool white LED array housed within the fluorometer optical head with each light step lasting for 4 min. At each light step, non-photochemical quenching (NPQ) was calculated following the conventional Bilger & Bjorkman [67] Stern-Volmer equation (see S1 Fig):

$$NPQ\;=\;{(F}_{m}-F_{m}\prime )\;/F_{m}\prime$$ (2)

where the prime (′) notation represents fluorescence measurements under actinic light. We used the approach of Serôdio *et al*. [68] where the maximum achieved value of *F*_m_′ throughout the FLC was used as a proxy for *F*_m_ in order to offset any down-regulation of fluorescence through dark-driven plastoquinone (PQ) pool reduction. However, values of NPQ determined through Eq 2 are unbounded, thus we calculated a complementary parameter that describes the yield of regulated non-photochemical quenching (YNPQ; see [16]), and generates a parameter bounded between 0 and 1:

$$YNPQ\;=\;(\frac{F\prime }{F_{m}\prime \;})-(\frac{F\prime }{F_{m}\;})$$ (3)

where *F*′ is the fluorescence measurement under actinic light and *F*_m_ was again taken as the maximum value of *F*_m_′ achieved throughout the FLC. Additional fluorescence yield parameters were further calculated following [16, 69] to describe the partitioning of absorbed excitation energy at PSII to include photochemical conversion (YII), non-regulated nonphotochemical quenching (YNO) to complement regulated nonphotochemical quenching (YNPQ) such that

YII+YNO+YNPQ=1 (4)

$$YII\;=\;(F_{m}^{\prime }-F\prime )/F_{m}^{\prime }$$ (5)

$$YNO\;=\;F'/F_{m}$$ (6)

To initially identify trade-offs in allocations of excitation energy to photochemical versus non-photochemical pathways, we also calculated the fraction of open RCIIs responsible for photochemical quenching [1-C] to plot vs. dynamic non-photochemical quenching [1-Q] under each actinic light intensity following equations from Suggett *et al*. [17] (Eqs 7 and 8). Both [1-C] and [1-Q] decrease in value from 1 to 0 with increasing extents of quenching, and the product of [1-C] and [1-Q] is equivalent to the PSII photochemical efficiency normalized to *F*_v_/*F*_m_,

$$\left[1-C]\;=\;(F_{m}\prime -F\prime )/(F_{m}\prime -F_{o}\prime \right)$$ (7)

$$\left[1-Q]\;=\;((F_{m}\prime -F_{o}\prime )/F_{m}\prime )/(F_{v}/F_{m}\right)$$ (8)

Where *F*_o_′ (fluorescence minimum under actinic light) is estimated as per Oxborough and Baker [70]:

$$F_{o}\prime \;=\;F_{o}/[(F_{v}/F_{m})\;+\;(F_{o}/F_{m}\prime )]$$ (9)

Measurements were performed for each species using at least three independent samples collected during acclimated exponential growth. Methods described from this point onwards apply to only the three selected diatoms (*Thalassiosira pseudonana*, *Thalassiosira oceanica*, *Thalassiosira weissflogii*) that span highly divergent photophysiological responses.

### Growth and biomass

Small aliquots from each culture were preserved daily (within 2 hours illumination) with glutaraldehyde (25%, Sigma-Aldrich) for later cell counting on a flow cytometer (CytoFlex S, Beckman Coulter, Miami, FL USA). Samples were counted for 60 s at a rate of 30 μL min^-1^. Specific growth rates (divisions [day]^-1^) were calculated as μ = ln(N_2_/N_1_)/(t_2_-t_1_), where μ is the specific growth rate (day^-1^), N_1_ and N_2_ are the cell concentrations (mL^-1^) at time 1 (t_1_) and time 2 (t_2_), respectively. Cell volume was calculated from the same sample used for cell count determinations using shape-specific geometric formulas from Sun & Liu [71] via an imaging compound light microscope (Nikon). From independent cultures, 10 images were taken at random and cellular dimensions recorded for five cells per image using Infinity software (Lumenera Corporation, Ontario, Canada).

On sampling days, two aliquots each of 5–8 mL culture were filtered onto a 25 mm glass fiber filter (Whatman GF/F) and stored overnight at -20°C in 90% acetone to extract chlorophyll *a*. Absorption was measured using a spectrophotometer (Aligent Technologies, Cary 60 UV-Vis) set at wavelengths 630, 657, 664, and 750 nm and chlorophyll *a* concentration was calculated according to Ritchie [72]. Cellular particulate organic carbon (POC, units of pg C mL^-1^) was also measured in duplicate for each biological replicate, by filtering two aliquots each of 5 mL onto pre-combusted filters (Whatman GF/F). POC samples were stored at -20°C until analysis on an elemental analyzer (LECO, Baulkham Hill, Australia) using culture filtrate (5 mL) as the blank. Net primary productivity (NPP) was calculated as the product of specific growth rate (μ) and cellular POC (C), whereby NPP = μ · C. Contributions from DOC were assumed to be negligible (<5% of total energy budget) for diatom cultures (*T*. *pseudonana*, [57]).

### PSII photo-inactivation and repair

Two subsamples (25 mL each) were initially collected from cultures. Using approaches adopted from Campbell *et al*. [32], lincomycin hydrochloride (95%, Sigma-Aldrich), an inhibitor of chloroplast protein synthesis, and thus PSII repair, was added to one subsample to a final concentration of 500 μg mL^-1^ while the second subsample (control) received no lincomycin hydrochloride addition [73]. After an initial dark incubation (10 min) of both subsamples to allow incorporation of inhibitor, photophysiology was repeatedly tracked via FRRf. Over a subsequent 120 min incubation at 1200 μmol photons m^-2^ s^-1^, 2 mL of fresh sample was taken for FRRf measurements at 30, 60 and 120 min. Each of these FRRf samples received consecutive induction flashlets every 10 s for a duration of 10 min without actinic light to capture short-term recovery of the PSII photochemical efficiency (*F*_v_′/*F*_m_′) resulting from relaxation of non-photochemical quenching as opposed to slower recovery from photo-inactivation. After the 120 min incubation at 1200 μmol photons m^-2^ s^-1^, subsamples were transferred to low light (~15 μmol photons m^-2^ s^-1^) for a total of 60 min to capture recovery. Recovery at 15 μmol photons m^-2^ s^-1^ was used instead of complete darkness since diatom PSII repair is stimulated by light intensities well below photosynthetic saturation [74, 75]. Measures of FRRf from five induction flashlets taken at 10 s intervals were completed after 30 and 60 min, hereafter referred to as 150 and 180 min, respectively, for consistency in assessment of this time-course experiment. Estimation of photo-inactivation and recovery of PSII were plotted from changes in *F*_v_′/*F*_m_′ or *F*_v_/*F*_m_ following each treatment time point.

Values of *F*_v_′/*F*_m_′ or *F*_v_/*F*_m_ from the sub-cultures treated with lincomycin and exposed to 1200 μmol photons m^-2^ s^-1^ (4 time points over 120 min) were fit with exponential decay curves to estimate the apparent first order rate constant for photo-inactivation of PSII under applied irradiance, k_PI_ (s^-1^). From k_PI_ we then calculated the susceptibility to photo-inactivation generalized across irradiance levels, as a target size functional absorption cross section for photo-inactivation of PSII, σ_I_ = k_PI_/photons m^-2^ s^-1^. The apparent first order rate constant for PSII repair, k_REC_ (s^-1^) [76] was then estimated following [13, 77, 78] using subsamples without lincomycin, across the entire treatment trajectory of initial exposure to 1200 μmol photons m^-2^ s^-1^ (4 time points) and then recovery at 15 μmol photons m^-2^ s^-1^ (2 time points). For fitting of k_REC_ we used the σ_I_ value for each species determined in the presence of lincomycin, on the simplifying assumption that the primary photo-inactivation of PSII is the same in the absence or the presence of PSII repair. Within each species each replicate time-course followed a similar trajectory and therefore k_PI_ and k_REC_ were fit using points pooled from 3–4 replicate trajectories for each species, using the nlsLM fitting function from the minpack.lm package [79] running under R [80] and RStudio [81]. Figures were then generated using ggplot2 [82].

### Pigment analysis

High performance liquid chromatography (HPLC) was used to determine concentrations of XC pigments (chlorophyll *a* and *c*, fucoxanthin, diadinoxanthin (Dd), diatoxanthin (Dt), and beta-carotene) in the diatom cultures. In triplicate for each species, 50 mL of culture were incubated in a 20°C waterbath at growth irradiance (Ig, 85 μmol photons m^-2^ s^-1^) and high light (HL, 1200 μmol photons m^-2^ s^-1^) for 10 min. Culture was then immediately filtered onto a GF/F filter at volumes to saturate the filter with material (25–40 mL) and thus maximize biomass for pigment signal detection. Filters were flash frozen in liquid nitrogen and stored at -80°C until extraction. Extraction of samples were carried out following Heukelem and Thomas [83] with slight modifications. Filters were placed into 15 mL tubes containing chilled acetone, sonicated (30 s) and then vortexed for 30 s (x3) under cold, dark conditions to limit pigment degradation, and then stored at 4°C overnight. Pigment extracts were then filtered through a 0.2 μM PTFE 13 mm syringe filter and stored in -80°C until analysis. An Agilent 1290 HPLC system equipped with a binary pump with integrated vacuum degasser, thermostatted column compartment modules, Infinity 1290 autosampler and PDA detector was used for the analysis. Column separation was performed using an Agilent’s Zorbax Eclipse XDB C8 HPLC 4.6 mm × 150 mm and guard column using a gradient of TBAA: Methanol mix (30:70) (solvent A) and Methanol (Solvent B) as follows: 0–22 min, from 5 to 95% B; 22–29 min, 95% B; 29–31 min, 5% B; 31–40 min, column equilibration with 5% B. Column temperature was maintained at 55°C. A complete pigment spectrum from 270 to 700 nm was recorded using PDA detector with 3.4 nm bandwidth. The de-epoxidation state (DPS) of XC pigments, particularly diadinoxanthin (Dd) de-epoxidation and diatoxanthin (Dt) epoxidation was determined as Dt (Dd-Dt)^-1^ [84] as diatoms have a dominant Dd-Dt cycle and minor violaxanthin-antheraxanthin-zeaxanthin cycle [46, 85, 86], compared to the dominant violaxanthin-antheraxanthin-zeaxanthin cycle in green algae and vascular plants [10, 87, 88].

### Membrane inlet mass spectrometry (MIMS)

Aliquots of 60 mL of culture were sparged with N_2_ gas for 20 min to remove ^16^O_2_, and 50 mL of the sparged sample was then transferred to a gas-tight syringe and directly enriched with labelled oxygen (^18^O_2_, Marshall Isotopes Ltd., Israel) and mixed vigorously by shaking for 3 min to allow the ^18^O_2_ gas bubble to equilibrate with the solution. ^18^O_2_-Labelled culture was then divided between four 12 mL exetainer vials (LabCo Ltd., UK) for the following treatments: time zero (T_0_), dark, growth irradiance (Ig, 85 μmol photons m^-2^ s^-1^) and high light (HL, 1200 μmol photons m^-2^ s^-1^). The T_0_ samples were fixed immediately with 200 μL 0.2 M mercuric chloride (HgCl_2_) to cease biological activity. Ig and HL vials were incubated under the specified light intensity for 20 min at 20°C then subsequently fixed in the same manner as T_0_. Fixed samples were stored at room temperature under darkness for later analysis on a membrane inlet mass spectrometer (MIMS, Bay Instruments, Maryland, USA).

Set up and analysis using the MIMS was undertaken following Kana *et al*. [56] modified by Suggett *et al*. [89]. In brief, samples were pumped through stainless steel capillary tubing, submerged in a waterbath (20°C), then over a semi-permeable microbore silicone membrane (Silastic^®^, DuPont), where gas exchange occurred. Gases flowed through a U-shaped manifold membrane inlet system, resting in a liquid N_2_ cryotrap, and attached to a Prisma QMS-200 (Pfeiffer) quadrapole mass spectrometer with a closed ion source and electron multiplier detector for recording mass/charge (m/z) ratios of 32 (^16^O_2_), 36 (^18^O_2_), and 40 (Ar). Discrete measurements of ion currents were recorded using QuikData software (Bay Instruments, Maryland, USA). Calibration of the MIMS was performed at the beginning and end of sampling and subsequently (~30 min) between sampling.

Rates of oxygen production/consumption were calculated from the difference between signal outputs, ^16^O_2_ and ^18^O_2_, for T_0_ and light treatment (Dark, Ig, HL) incubation samples before scaling to hourly rates (as per [89]). Corrected ^16^O_2_ signals in the light were assumed to be gross oxygen production (GP_O2_). Net oxygen production (Net_O2_) was calculated as the difference between GP_O2_ and total respiration, including dark (R_DARK_) and light dependent respiration (LDR). R_DARK_ is the ^18^O_2_ signal from the dark sample and LDR is the difference between R_DARK_ and ^18^O_2_ signal from each light treatment (Ig or HL). It is important to note that studies with continuous (i.e. real-time) MIMS sampling [14, 90–93] are able to establish highly accurate rates of oxygen consumption/production, whereas discrete measurements [89, 94, 95]–and as per our study–likely underestimate ‘true’ rates. A 20 min incubation was chosen to minimize the ^16^O_2_ consumed by respiration while allowing enough time for generation of detectable oxygen signals via MIMS. Without real-time rate information (i.e. continuous measuring) it is impossible to determine when ^16^O_2_ exceeds ^18^O_2_ and therefore a portion of this ^16^O_2_ signal is likely to be consumed; as such, GP_O2_ is thus likely an underestimate of true ^16^O_2_ production. However, by this justification, ^18^O_2_ consumption (respiration) is also likely an under- (conservative) estimate of true values. Having this consistency in assessing the values derived from MIMS provided confidence that the trends observed are accurate; however, the true concentration values may be underestimated. Due to the sensitive nature of MIMS sampling, at least five replicates (and two measurements per replicate) were collected for each species.

### Statistics

Differences in cellular properties between species were assessed using one-way analysis of variance (ANOVA) followed by Bonferroni’s multiple comparison test where prerequisite assumptions of normality and homoscedasticity were satisfied (tested for using Levene’s and Shapiro-Wilk tests respectively). If the assumption of normality was violated, data were either square-root or arcsine-square-root transformed and the distribution of residuals re-tested. If either assumption continued to be violated despite transformation, differences between species were instead evaluated using non-parametric ANOVA on ranks (Kruskal-Wallis test), followed by Dunn’s post-hoc test. Two-way ANOVA followed by Bonferroni’s multiple comparison test was used to evaluate the significance of the effect of species and light treatment on photobiological characteristics (pigments and DPS) [96]. Residuals of all variables exhibited normal distribution, although assumptions of homoscedasticity were violated for specific variables: Dd, Dt, GP_O2_, Net_O2_ and LDR. ANOVAs were performed using IBM SPSS Statistics v26, while Sigmaplot v12.5 was used for data transformation and Kruskal-Wallis tests. As the variable YNPQ exhibited neither normal distribution or equal variance across sample groups, a permutation univariate ANOVA was performed using the PERMANOVA+ add-on [97] in the PRIMER (v6) statistical package (PRIMER-E Ltd, UK). A resemblance matrix computed from Euclidean distance was used for the PERMANOVA procedure, and the test comprised a two-factor design (species and light treatment), type I (sequential) sum of squares and 9999 permutations under the reduced model. The significance level for all tests performed was set at *p* < 0.05.

## Results

### Initial photophysiological assessment

**Fig 1:**
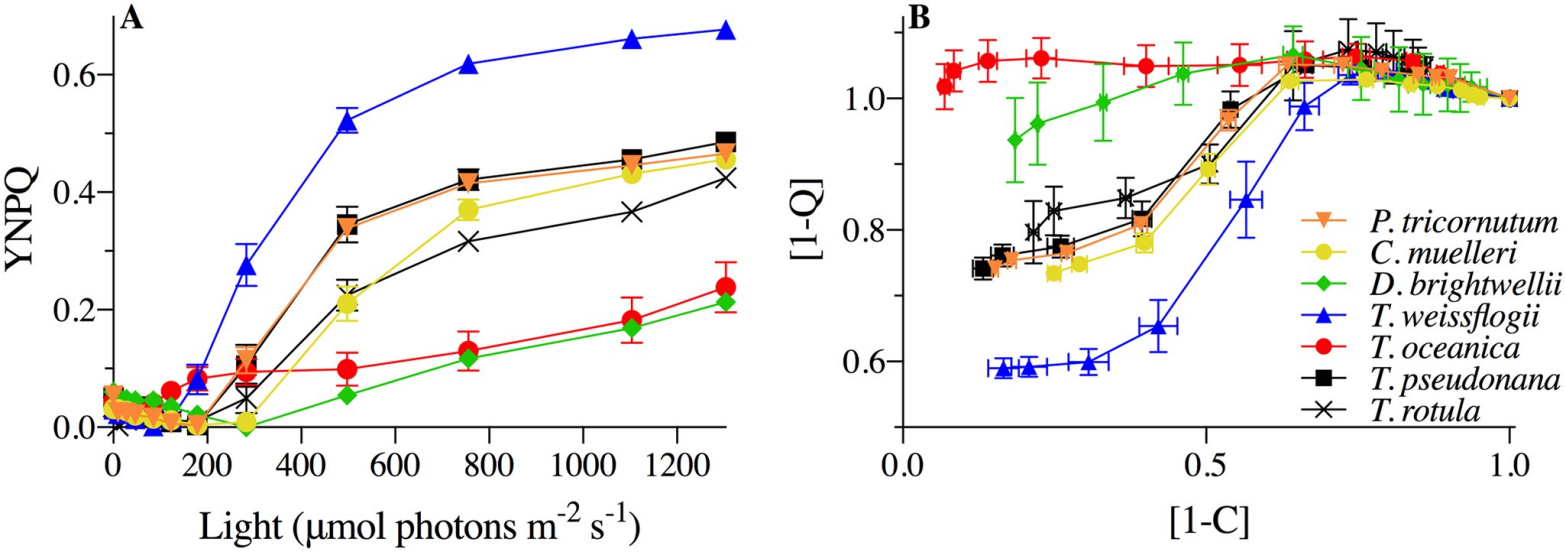
Initial photophysiological monitoring of seven diatoms: *Phaeodactylum tricornutum* (orange inverted triangles), *Chaetoceros muelleri* (yellow circles), *Ditylum brightwellii* (green diamonds), *Thalassiosira rotula* (black Xs), *Thalassiosira pseudonana* (black squares), *Thalassiosira weissflogii* (blue triangles), and *Thalassisosira oceanica* (red circles). (A) Yield of non-photochemical quenching (YNPQ; see Eq 3) with increasing light intensity. (B) Dynamic non-photochemical quenching [1-Q] versus photochemical [1-C], where data points signify responses to stepped increases in light intensity starting from 0 (far right point) to 1304 μmol photons m^-2^ s^-1^ (far left point) for 4 min at each light intensity. Error bars represent the standard error of the mean of at least *n* = 3 for independent biological replicates.

Excitation allocation strategies were investigated in diatoms species with different ecological niches; specifically, the coastal pennate *Phaeodactylum tricornutum*, coastal small centric *Chaetoceros muelleri*, coastal large centric *Ditylum brightwellii*, and four centric species of the *Thalassiosira* genus ranging in cell size from smallest to largest: coastal *T*. *pseudonana*, open ocean *T*. *oceanica*, esturaine *T*. *weissflogii*, coastal *T*. *rotula*. Cells were grown under 85 μmol photons m^-2^ s^-1^ and FRRf-response light curves were used to determine YNPQ (Eq 3, Fig 1A) and the proportion of energy directed to [1-C] (photochemistry, Eq 7) versus [1-Q] (photoprotection, Eq 8) (Fig 1B). The taxa fell into three excitation allocation strategies: (i) relatively fast/substantial, (ii) relatively slow/negligible accumulation of nonphotochemical quenching vs. declines of [1-Q], and (iii) an intermediary response.

*T*. *weissflogii*, *T*. *oceanica* and *T*. *pseudonana*, were chosen as representative candidates of the three strategies observed to subsequently examine the diversity of mechanisms utilized by these related diatoms under dynamic light to maintain photosynthetic efficiency. Under the stepwise progression of increasing actinic light intensity, *T*. *oceanica*, the open ocean species, initiated negligible nonphotochemical quenching (parameterized as YNPQ, Fig 1A). In contrast, *T*. *weissflogii*, an estuarine native species, rapidly initiated nonphotochemical quenching at relatively low light intensity (~120 μmol photons m^-2^ s^-1^) and to a higher capacity. *T*. *pseudonana*, a coastal species, exhibited an intermediate response, initiating nonphotochemical quenching once reaching a light intensity of ~200 μmol photons m^-2^ s^-1^. A similar pattern was observed when comparing [1-C] and [1-Q] (Fig 1B). Here [1-Q] showed limited short-term change in response to a progressive decrease in [1-C] (RCII closure) under increasing light in *T*. *oceanica*. In contrast *T*. *weissflogii* showed strong induction of [1-Q] as [1-C] dropped below 0.6 with increasing light. Values greater than 1 for [1-Q] are attributed to the effects of chlororespiration under very low light/darkness [46, 68]. Overall, increased reliance on non-photochemical quenching generally corresponded with decreased photochemical quenching, tracking the remaining fraction of RCIIs engaged in photochemistry, across these three species.

### Cell characteristics for selected *Thalassiosira* species

Cellular growth rates increased with decreasing cell size from *T*. *weissflogii* to *T*. *pseudonana* (Table 1). Chl *a*:C and C:N were not statistically different among the three species and are consistent with previous reports for *T*. *pseudonana* [57], *T*. *weissflogii* (Chl *a* and μ, [98, 99]), and *T*. *oceanica* [100]. Net primary production (NPP in units of μmol C (mg Chl h)^-1^) in *T*. *oceanica* and *T*. *pseudonana* were not statistically distinguishable from NPP in *T*. *weissflogii* (Table 1). Consistent with previous reports for diatoms grown under steady-state nutrient replete conditions, values of FRRf-based maximum photochemical efficiency (*F*_*v*_*/F*_*m*_, dimensionless) were generally higher (and the effective absorption cross section for PSII photochemistry, σ_PSII_, smaller) for the larger *T*. *weissflogii* compared to the smaller *T*. *oceanica* and *T*. *pseudonana* [101].

**Table 1:** Cell characteristics of *Thalassiosira weissflogii*, *Thalassiosira oceanica*, and *Thalassiosira pseudonana* from exponential steady state growth under 85 μmol photons m^-2^ s^-1^ at 20°C.

Species	Growth rate (d^-1^)	Cells mL^-1^ (x10^5^)	Cell volume (μm^3^)	Chl *a* cell^-1^ (pg)	C cell^-1^ (pg)	Chl:C (x10^-2^) (μg μg^-1^)	C:N	NPP (μmol C (mg Chl h)^-1^)	*F*_v_/*F*_m_	σ_PSII_ (nm^2^ PSII^-1^)
***Thalassiosira weissflogii***	0.68 (0.05)	1.40^a^ (0.03)	1243.10^b^ (62.26)	5.66^b^ (0.20)	158.91^b^ (23.24)	4.32 (0.17)	3.82 (0.13)	67.62 (16.91)	0.58^b^ (0.01)	2.29^a^ (0.16)
***Thalassiosira oceanica***	0.80 (0.02)	3.62^a^ (0.18)	159.62^a^ (19.38)	1.03^a^ (0.01)	56.13^a^ (3.23)	2.92 (0.48)	5.23 (0.87)	88.36 (17.94)	0.55^a^ (0.00)	3.98^b^ (0.17)
***Thalassiosira pseudonana***	0.94 (0.03)	12.68^b^ (0.27)	99.82^a^ (1.77)	0.60^a^ (0.03)	7.92^a^ (0.70)	4.11 (0.35)	4.68 (0.80)	84.41 (13.62)	0.56^a^ (0.00)	3.01^a^ (0.21)
ANOVA or KW	H = 5.4	H = 9.8	F = 601.0	F = 46.6	F = 28.1	F = 5.1	F = 1.2	H = 0.6	F = 34.9	F = 17.2
(F/H, *p*)	*p*>0.05	***p*<0.05**	***p*<0.05**	***p*<0.05**	***p*<0.05**	*p*>0.05	*p*>0.05	*p*>0.05	***p*<0.05**	***p*<0.05**

Data shown are the mean of independent biological replicates where n = 3 for growth rate (d^-1^), Chl *a* (cell^-1^) and net primary production (NPP), n = 4 for cell concentration (mL^-1^), carbon (pg cell^-1^), C:N, *F*_v_/*F*_m_ and σ_PSII_ (nm^2^ PSII^-1^) and n = 20 for cell volume. Values in parentheses are SE of the mean. ANOVA or Kruskal-Wallis (KW) test results comparing species and individual cell characteristics are presented using F or H-values, respectively, with significant *p*-values (*p* < 0.05) in bold. Superscripted letters indicate differences between species groups identified by Bonferroni post-hoc analysis.

### Photophysiology strategy

Contents of accessory chlorophyll and carotenoids were normalized to Chl *a* and were largely similar for samples incubated for 10 min under HL (1200 μmol photons m^-2^ s^-1^) compared to those at the growth irradiance (Ig, 85 μmol photons m^-2^ s^-1^) (Table 2) within each species. Chl *c*, fucoxanthin and β-carotene, while not influenced by short-term high light (*p* > 0.05), were different among species (*p* < 0.05) with fucoxanthin:Chl *a* highest in *T*. *oceanica* compared to *T*. *pseudonana* and *T*. *weissflogii* (Table 2). Most notably, extent of XC, determined as the de-epoxidation state (DPS, see Methods *‘Pigment Analysis’*), differed between light treatments where all species exhibited an increase in DPS, (as a decrease in Dd:Chl *a*; and corresponding increase in Dt:Chl *a*) from Ig to HL. *T*. *pseudonana* exhibited the lowest DPS under Ig but also the greatest difference in DPS between Ig and HL (Table 2).

**Table 2:** Xanthophyll cycle pigments under growth irradiance (Ig) and transient short-term shift to high light (HL).

Species	Light	Normalized to Chl *a* (μg mL^-1^)					DPS
		Chl *c*	Fuc	β-Car	Dd	Dt	
***T*. *weissflogii***	Ig	0.028 (0.002)	0.533 (0.007)	0.021 (0.001)	0.034 (0.002)	0.012 (0.004)	0.243 (0.076)
	HL	0.027 (0.002)	0.525 (0.003)	0.021 (0.001)	0.026 (0.002)	0.028 (0.004)	0.509 (0.031)
***T*. *oceanica***	Ig	0.053 (0.002)	0.959 (0.016)	0.017 (0.001)	0.032 (0.001)	0.008 (0.001)	0.205 (0.013)
	HL	0.049 (0.005)	0.953 (0.034)	0.018 (0.000)	0.027 (0.004)	0.027 (0.007)	0.492 (0.080)
***T*. *pseudonana***	Ig	0.033 (0.005)	0.659 (0.006)	0.019 (0.000)	0.049 (0.007)	0.005 (0.003)	0.079 (0.031)
	HL	0.031 (0.004)	0.650 (0.008)	0.020 (0.000)	0.036 (0.004)	0.025 (0.011)	0.372 (0.072)

Diadinoxanthin (Dd), diatoxanthin (Dt), and accessory pigments, Chlorophyll *c* (Chl *c*), fucoxanthin (Fuc), and Beta-carotene (β-Car) were all normalized to Chl *a* for *T*. *weissflogii*, *T*. *oceanica*, and *T*. *pseudonana* under Ig (85 μmol photons m^-2^ s^-1^) and HL (1200 μmol photons m^-2^ s^-1^) incubation for 10 min. Data averaged from 3 independent replicates. Value in parentheses represent SE of the mean.

**Fig 2:**
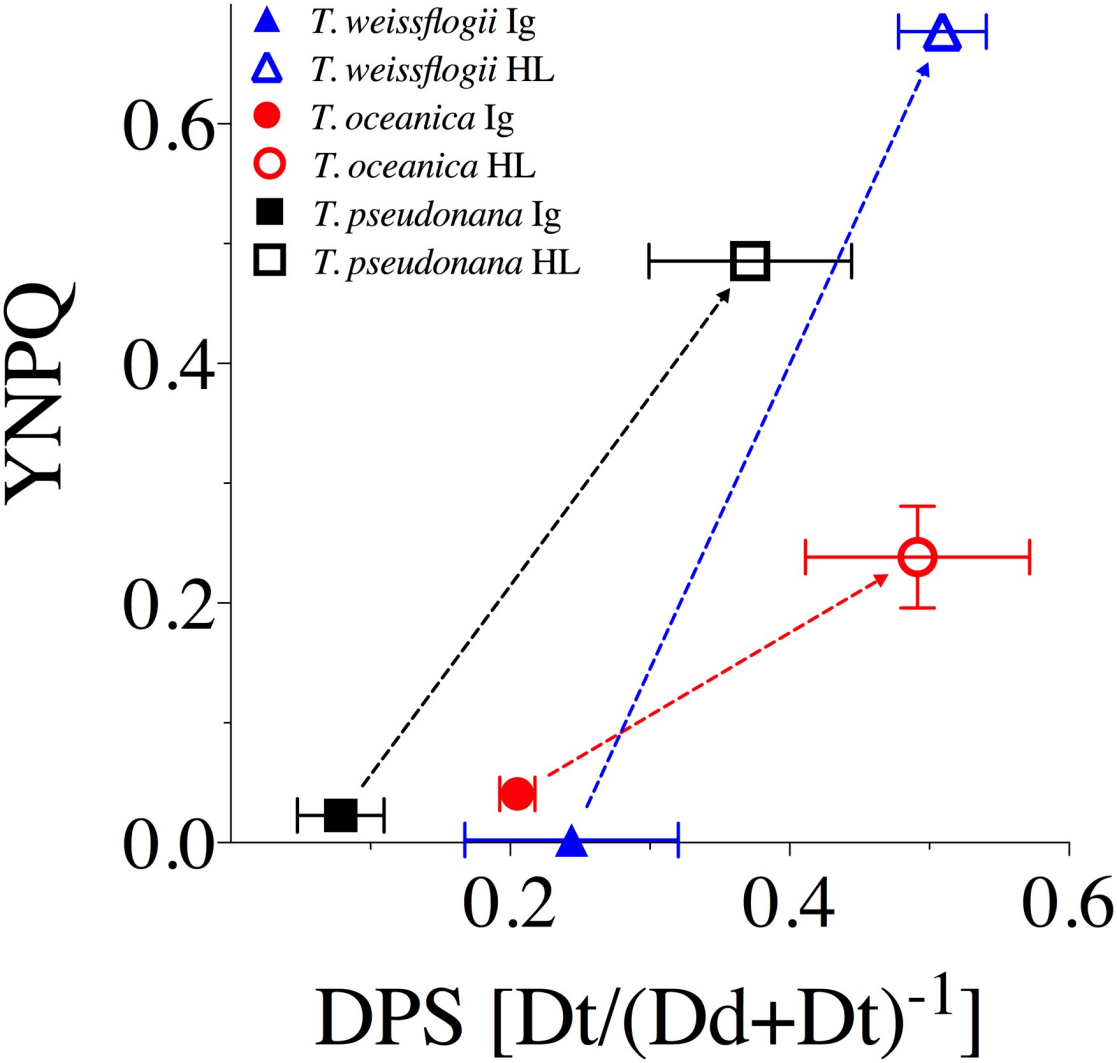
YNPQ vs. de-epoxidation state (DPS) of the XC for *T*. *weissflogii* (blue triangles), *T*. *oceanica* (red circles), and *T*. *pseudonana* (black squares) under 10 min exposure to growth irradiance (Ig, 85 μmol photons m^-2^ s^-1^, solid symbols) and high light (HL, 1200 μmol photons m^-2^ s^-1^, open symbols). Dashed lines highlight the extent of changes observed between light treatments for each measured parameter. Data averaged from three independent replicates for DPS and at least four independent replicates for YNPQ with errors bars representing SE of the mean.

Increases in DPS from Ig to HL generally accompanied increases in non-photochemical quenching (parameterized as YNPQ, Fig 2) for all species, but, importantly, the extent of DPS did not show comparable correlations with YNPQ across species. *T*. *weissflogii* exhibited much higher nonphotochemical quenching than *T*. *oceanica* (*p* < 0.001) despite similar DPS. Whilst *T*. *pseudonana* had lower total DPS, the corresponding increases of DPS and YNPQ from Ig to HL were more similar to those of *T*. *weissflogii* (dashed lines, Fig 2). Thus, the extent to which nonphotochemical quenching induction accompanied DPS accumulation appeared to differ among these three species, but was not explicitly tested in our analysis.

### PSII photo-inactivation and repair

**Fig 3:**
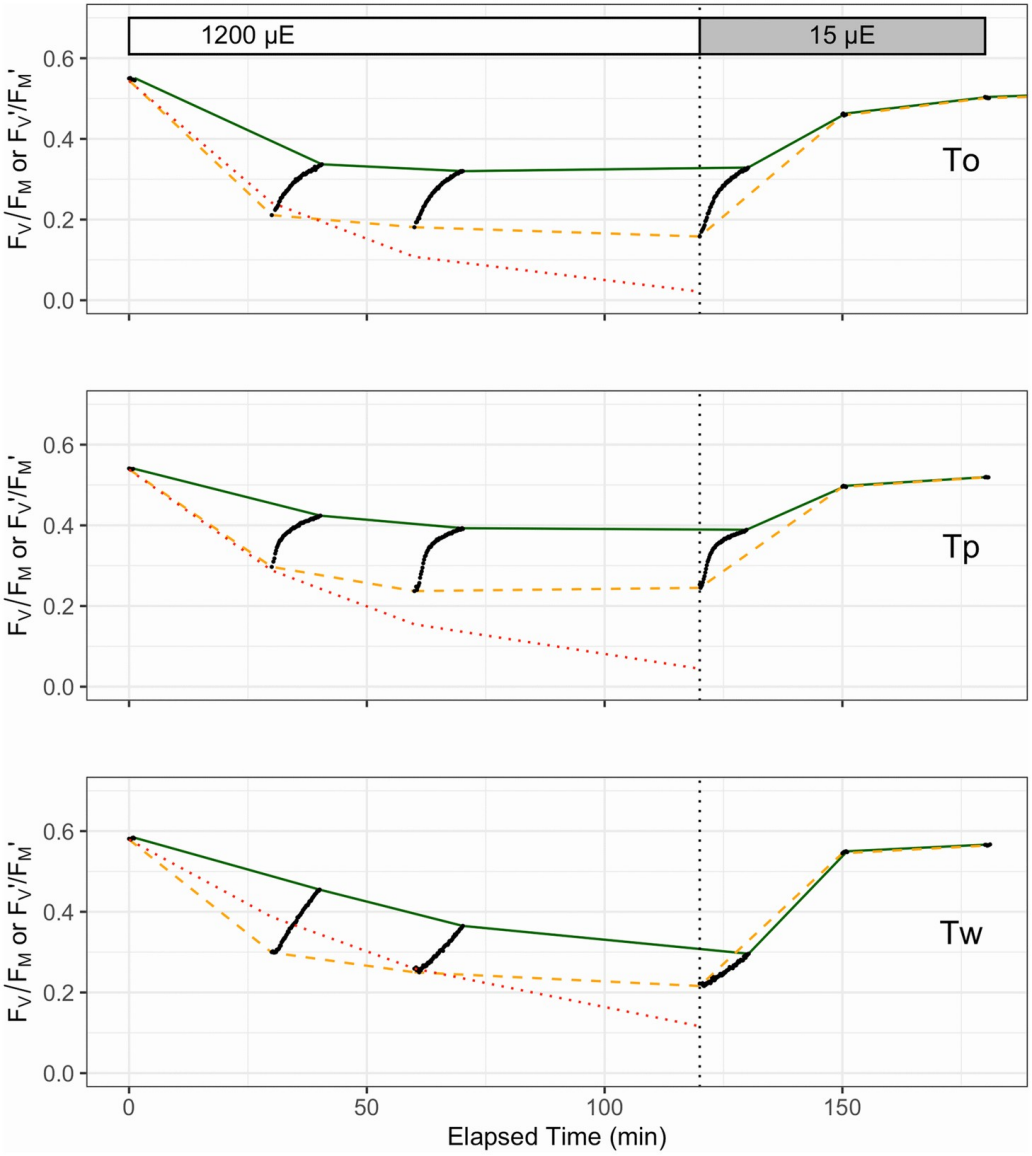
Representative photo-inhibition and recovery time courses for *T*. *oceanica* (To), *T*. *pseudonana* (Tp) and *T*. *weissflogii* (Tw). Black points show individual determinations of *F*_*v*_′/*F*_*m*_′ or *F*_*v*_*/F*_*m*_ from FRRf measurements of cultures with PSII repair active (absence of lincomycin). Dashed orange line connects *F*_*v*_′/*F*_*m*_′ measures taken immediately after exposure to HL (1200 μmol photons m^-2^ s^-1^) over 0–120 min or recovery light (15 μmol photons m^-2^ s^-1^) at 150 and 180 min, influenced by combined effects of non-photochemical quenching induction and net photo-inactivation (if any). Solid green line connects *F*_*v*_*/F*_*m*_ taken after 10 min of subsequent dark for HL time points, to allow relaxation of non-photochemical quenching, or taken immediately during the recovery light period. These points were used to fit the Kok model of PSII photo-inactivation countered by repair. Note the different patterns and amplitudes of short-term (10 min) relaxation of non-photochemical quenching, among the species (black dots). The dotted red line shows *F*_*v*_*/F*_*m*_ data from separate lincomycin treated cultures to show the underlying photo-inactivation in the absence of counteracting repair.

We examined the extent of PSII photo-inactivation and capacity for repair by exposing the cells to HL (1200 μmol photons m^-2^ s^-1^) for 120 min, followed by a recovery period at 15 μmol photons m^-2^ s^-1^ (Fig 3). All three species exhibited a drop in *F*_*v*_′/*F*_*m*_′ and *F*_*v*_*/F*_*m*_ over the initial 30–60 min of HL exposure. *T*. *oceanica* and *T*. *pseudonana* then stabilized *F*_*v*_′/*F*_*m*_′ and *F*_*v*_*/F*_*m*_, reflecting the induction of PSII repair. Upon the down-shift to15 μmol photons m^-2^ s^-1^ all species showed initial rapid recoveries, as photo-inactivation dropped to very low rates, and PSII repair continued for 30 min until all species had recovered to near-initial levels of *F*_*v*_*/F*_*m*_.

In parallel with the effects of photo-inactivation and repair, Fig 3 shows the kinetically overlapping influences of nonphotochemical quenching induction upon *F*_*v*_′/*F*_*m*_′ (orange dashed line) measured immediately after exposure to treatment or recovery light. Systematic photophysiological assessment of samples removed over the 120 min HL exposure and subsequently subjected to a 10 min dark period before fluorometric assessment highlighted the influence of nonphotochemical quenching on PSII function. This is particularly evident in *T*. *oceanica* and *T*. *pseudonana* where initial *F*_*v*_′/*F*_*m*_′ largely relaxed to higher *F*_*v*_*/F*_*m*_ (through relaxation of nonphotochemical quenching), tracked with the black dots connecting the orange dashed and green solid lines, respectively (see Fig 3), over the entire 120 min in HL. In contrast, *T*. *weissflogii* showed a progressive decrease in the relaxation amplitude over 120 min HL indicative of sustained (longer lived) nonphotochemical quenching, consistent with the failure of *T*. *weissflogii* to fully counter photo-inactivation through induction of repair [74]. It is thus difficult to discriminate between the kinetically overlapping processes of photo-inactivation and sustained downregulation of PSII.

Replicate time courses for PSII photo-inactivation in the presence of lincomycin were fit for each species to extract the rate constant for photoinactivation, k_PI_. These k_PI_ values determined for each species in the absence of PSII repair were then inputted into fit models [13, 76] using data captured from culture samples with PSII repair, in order to estimate the apparent first order rate constant for PSII repair, k_REC_ (Table 3). As expected [73], the susceptibility to photo-inactivation (k_PI_) decreased with increasing diatom cell size from the smaller *T*. *oceanica* and *T*. *pseudonana* to the larger *T*. *weissflogii* (Table 3). The rate constant for the counteracting PSII repair process (k_REC_) also decreased with increasing cell size from *T*. *pseudonana* to *T*. *weissflogii*, but was also low in the offshore *T*. *oceanica*, consistent with patterns of higher capacity for PSII repair observed previously for onshore versus offshore diatoms [32].

**Table 3:** Rate constants for PSII photo-inactivation (k_PI_) and repair (k_REC_) for *T*. *oceanica*, *T*. *pseudonana* and *T*. *weissflogii*, after acclimated growth at 85 μmol photons m^-2^ s^-1^, followed by treatment for 120 min under HL (1200 μmol photons m^-2^ s^-1^) then low light recovery (15 μmol photons m^-2^ s^-1^) (Fig 3).

Species	k_PI_ (s^-1^)	k_REC_ (s^-1^)
***T*. *oceanica***	4.50e-04 (2.28e-05)	5.48e-04 (6.68e-05)
***T*. *pseudonana***	3.46e-04 (1.30e-05)	1.50e-03 (1.35e-04)
***T*. *weissflogii***	2.23e-04 (9.14e-06)	3.16e-04 (3.35e-05)

Values derived from curve fits [13, 76] of data from 3 independent replicates. Values in parentheses represent SE of the mean.

**Fig 4:**
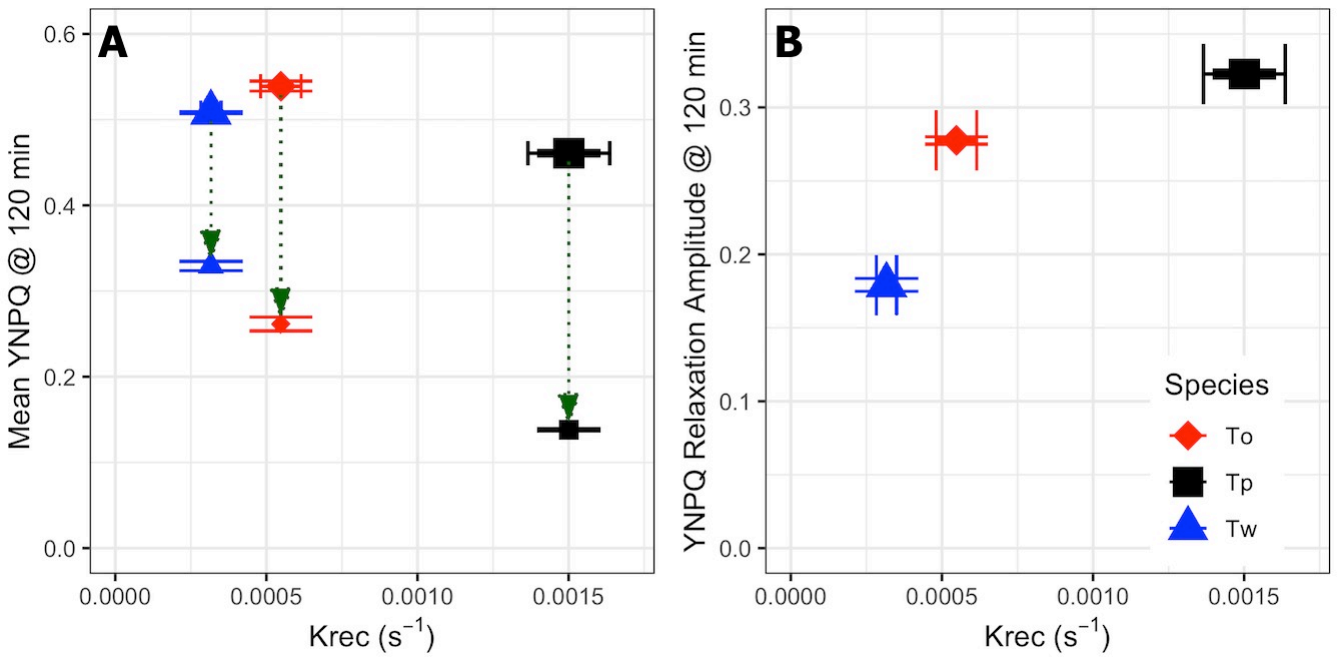
Mean YNPQ and YNPQ relaxation amplitude for *T*. *weissflogii*, *T*. *oceanica* and *T*. *pseudonana* after 120 min high light exposure. (A) Mean values of YNPQ vs. the rate constant for PSII repair, k_REC_, over 120 min HL exposure showing nonphotochemical quenching measured immediately after light exposure (larger symbols) or after 10 min dark FRRf incubation (smaller symbols) for *T*. *weissflogii* (Tw, blue triangles), *T*. *oceanica* (To, red diamonds) and *T*. *pseudonana* (Tp, black squares). (B) The amplitude of YNPQ relaxation (also green arrows in (A)). Error bars show standard errors of the estimates for 3 or 4 independent biological replicates.

The extent of nonphotochemical quenching induction after 120 min of HL, and then relaxation over the subsequent 10 min dark period, were next compared with the PSII repair rates. Interestingly, all species started with similar values of induced YNPQ of ~0.45–0.5 (Fig 4) under the high light treatment. This contrasts with the much lower values of YNPQ observed for *T*. *oceanica* compared to *T*. *weissflogii* from shorter incubations (Figs 1 and 2), highlighting that *T*. *weissflogii* rapidly builds a rapid but sustained nonphotochemical quenching whereas *T*. *oceanica* more slowly builds nonphotochemical quenching over time. However, differences in YNPQ between the beginning and end of the 10 min dark incubation (Fig 4A), and hence the amplitude of nonphotochemical quenching relaxation (Fig 4B), increased with PSII repair capacity. Thus, *T*. *pseudonana* had the highest PSII repair capacities and maintained a large amplitude of nonphotochemical quenching relaxation whereas *T*. *oceanica* and, particularly, *T*. *weissflogii* show sustained nonphotochemical quenching (Fig 4) albeit with slower induction for *T*. *oceanica* (Fig 1).

### Light-driven O_2_ consumption

**Fig 5:**
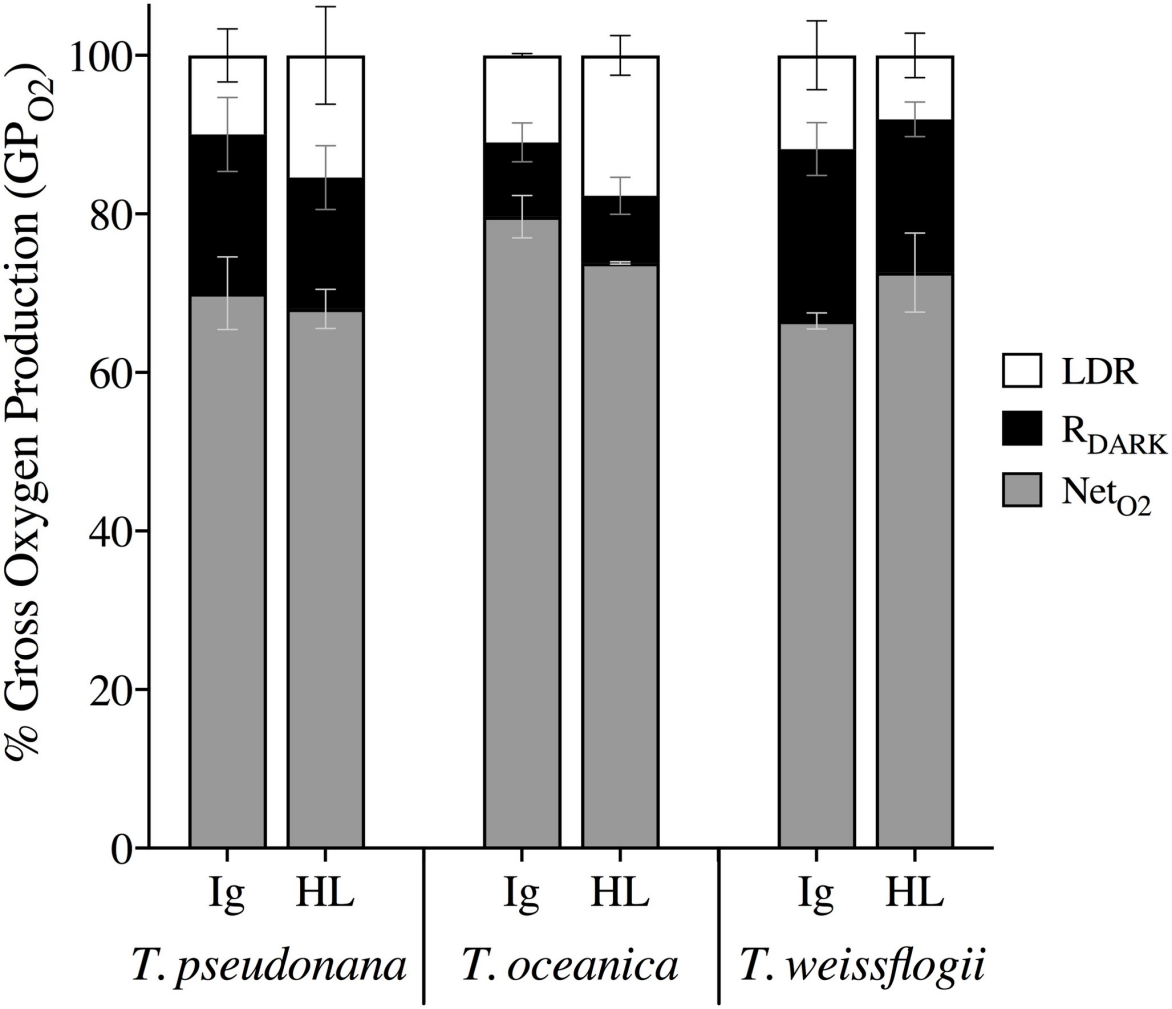
Proportions of total photochemical energy (GP_O2_) allocated to various oxygen pathways over a 20 min incubation under growth irradiance (Ig) and high light (HL). Fractional percentages of GP_O2_ included net oxygen production (Net_O2_, grey), dark respiration (R_DARK_, black) and light dependent respiration (LDR, white) in *T*. *weissflogii*, *T*. *oceanica*, and *T*. *pseudonana* under Ig (85 μmol photons m^-2^ s^-1^) and HL (1200 μmol photons m^-2^ s^-1^). Data averaged from 2 or 3 independent biological replicates with error bars representing SE of the mean.

Cell normalized measurements of gross O_2_ production (GP_O2_; pmol cell^-1^ h^-1^) via MIMS were consistent across light treatments where there was higher production under HL than Ig for all three species (*p* < 0.05, S1 Table). However, *T*. *weissflogii* GP_O2_ was significantly higher from both *T*. *oceanica* and *T*. *pseudonana* (Bonferroni post-hoc *p* < 0.0001). Measurements of energy losses are shown in Fig 5 as the fraction of GP_O2_ allocated to respiration in the dark (R_DARK_) and in the light (LDR), with the remainder being net O_2_ production (Net_O2_). Extent of LDR (% of GP_O2_) was comparable for all species under Ig but not HL; specifically, %LDR for *T*. *weissflogii* decreased from 11.8% at Ig to 8.1% at HL but increased from an average of 10.5% at Ig to 16.6% at HL for the other two species, with *T*. *oceanica* exhibiting the highest LDR increase from Ig to HL. Patterns of %R_DARK_ were more variable across species and treatment, whereby %R_DARK_ in *T*. *weissflogii* and *T*. *pseudonana* was 16.6–21.7% regardless of irradiance and %R_DARK_ in *T*. *oceanica* was always <10% (Fig 5, S2 Table). This trend was supported by Fisher’s Tukey post-hoc analysis where *T*. *oceanica* differed from both *T*. *weissflogii* (*p* = 0.017) and *T*. *pseudonana* (*p* = 0.033; see S2 Table for full comparisons). Together, the trade-offs in %LDR and %R_DARK_ from Ig to HL for *T*. *pseudonana* resulted in unchanged %Net_O2_, whereas the light-dependent changes in %LDR (but not %R_DARK_) resulted in an increase and decrease of Net_O2_ from Ig to HL for *T*. *weissflogii* and *T*. *oceanica*, respectively (Fig 5).

### Energy excitation fluxes

**Fig 6:**
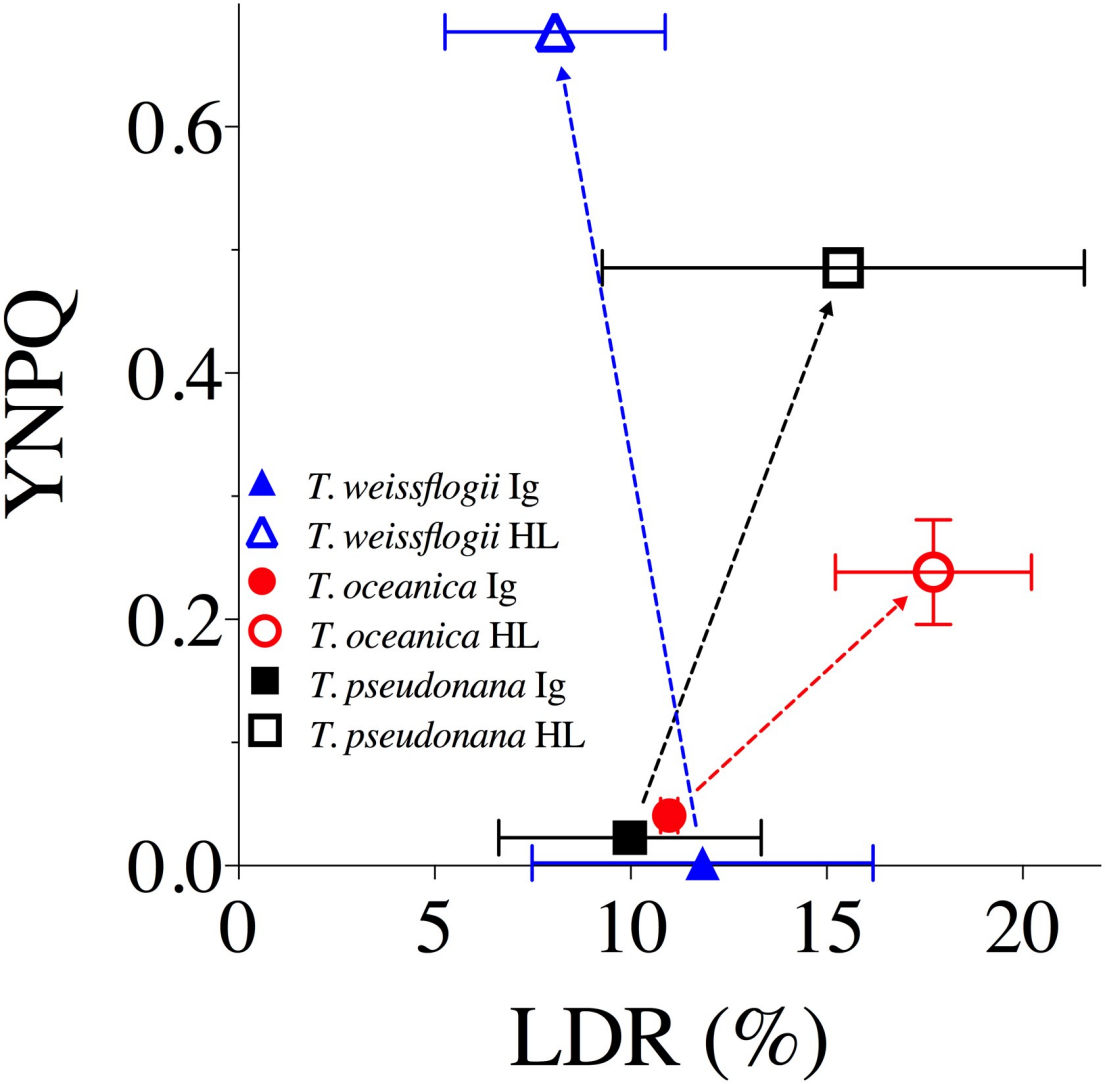
The yield of non-photochemical quenching (YNPQ) versus light dependent respiration (LDR) as a % of GP_O2_ for *T*. *weissflogii* (blue triangles), *T*. *oceanica* (red circles), and *T*. *pseudonana* (black squares) under 20 min exposure to growth irradiance (Ig, 85 μmol photons m^-2^ s^-1^, solid data points) and high light (HL, 1200 μmol photons m^-2^ s^-1^, open data points). Data averaged from 2 or 3 independent replicates for LDR and at least 4 independent replicates for YNPQ. Error bars represent SE of the mean.

No variation in %LDR or YNPQ was evident amongst species at Ig; however, %LDR at HL exhibited a negative correlation with YNPQ across the three species (Fig 6), where *T*. *oceanica* exhibited the highest %LDR despite low capacity for nonphotochemical quenching thus complementing the highest k_PI_ rates observed under HL (Table 3). *T*. *weissflogii* displayed the opposite response with the lowest reliance on LDR and highest YNPQ (Fig 6) supporting lowest k_REC_ and k_PI_ rates observed compared to other species (Table 3).

**Fig 7:**
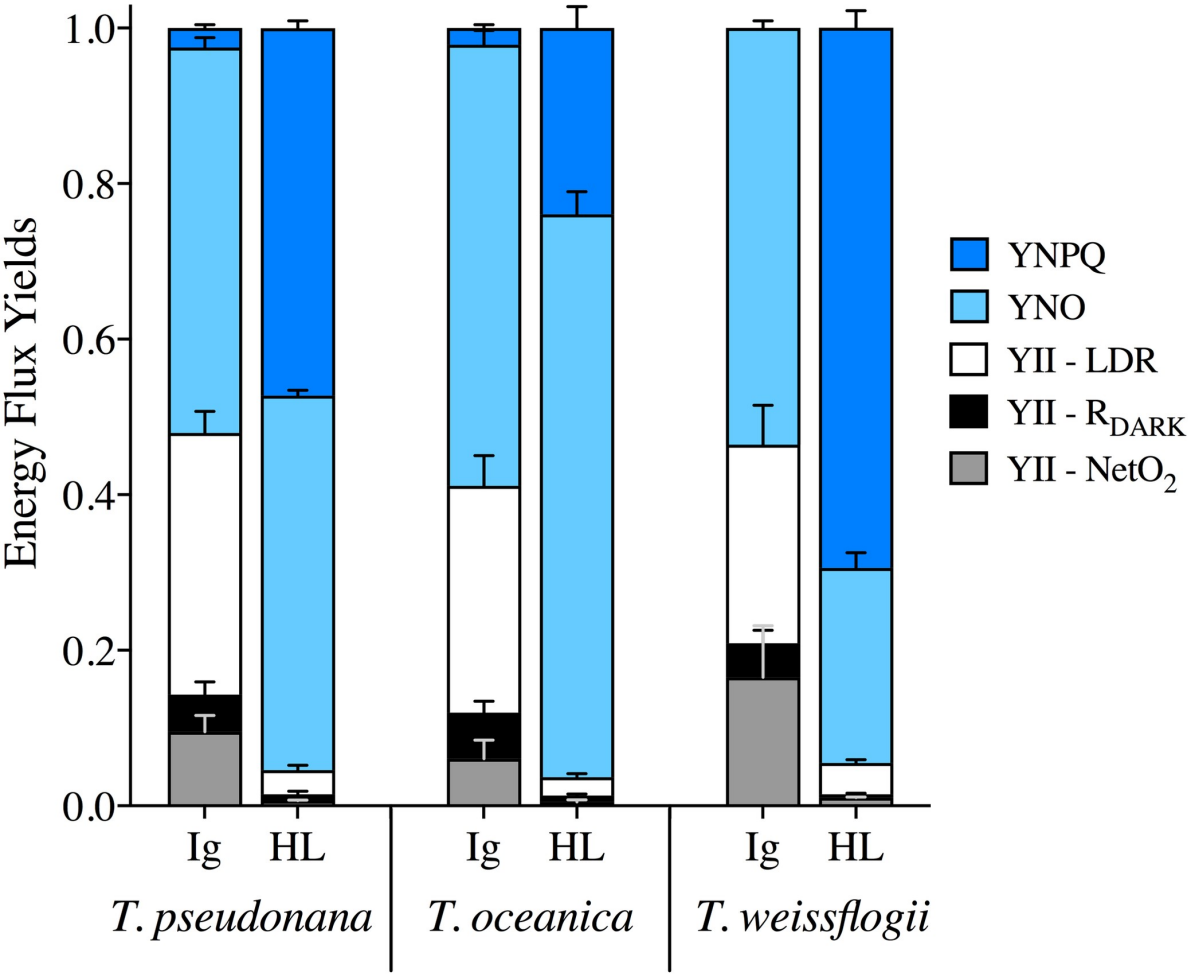
Energy flux yields. YNPQ (dark blue), YNO (light blue) and YII, which is then further divided into fractions of LDR (white), R_DARK_ (black) and Net_O2_ (grey), from *T*. *pseudonana*, *T*. *oceanica* and *T*. *weissflogii* under 85 μmol photons m^-2^ s^-1^ (Ig) and 1200 μmol photons m^-2^ s^-1^ (HL). Data averaged from 3 independent biological replicates and error bars represent SE of the mean.

The collective energy loss yields via YII, YNPQ and YNO (as per Eq 4) revealed clear differences across the species (Fig 7). At Ig, the proportion of all absorbed light allocated to photochemical conversion (parameterized as YII, Eq 5) was 0.4–0.45; although in *T*. *pseudonana* YII is significantly higher than in *T*. *oceanica* (ANOVA for YII across species, *p* = 0.022; Bonferroni post-hoc, *p* = 0.03). Remarkably, for all species under HL only 0.05 of the fluorescence-derived yields for absorbed light energy went to YII (Fig 7) (ANOVA, *p* = 0.236), which contradicts [102] showing higher fluxes via pseudo-cyclic electron flow in diatoms. There is a greater proportion of energy flux via YNPQ for *T*. *weissflogii* (~0.7) under HL while, instead, YNO represents ~0.7 of excitation flux in *T*. *oceanica*. *T*. *pseudonana* has balanced YNO and YNPQ at ~0.48 each, once again revealing the divergence of photoprotective strategies employed by these diatoms. Since FRRf-based values of YII appear to, generally, be directly proportional to GP_O2_ in microalgae [e.g. 103], we further considered the %LDR, %R_DARK_ and %Net_O2_ allocations of GP_O2_ to be comparable to YII (Fig 7). This demonstrates that a very small fraction of absorbed light ultimately results in ‘O_2_ recycling’ (loss of electrons in the membrane electron transport chain) through %LDR across these diatom species; specifically, 4–6% under Ig and <1% under HL.

## Discussion

**Fig 8:**
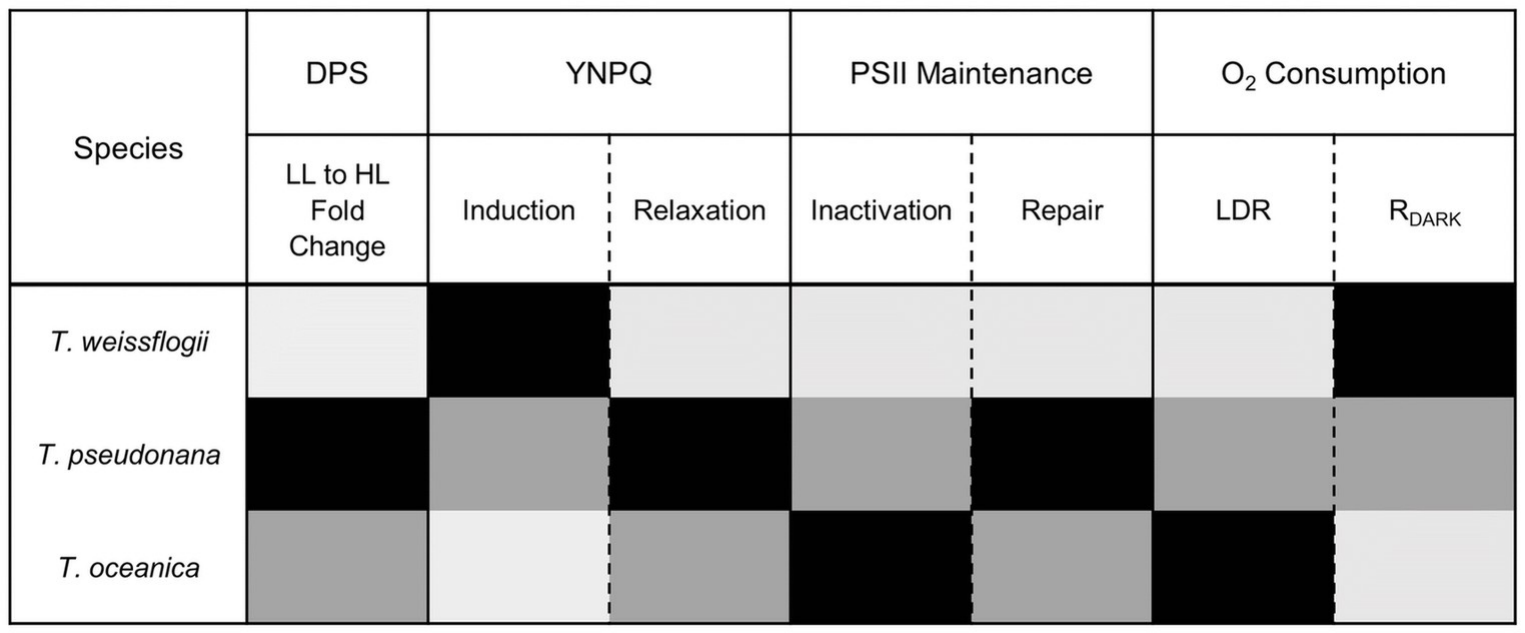
Summary of relative reliance (low to high; light grey to black) on various energy dissipation strategies when subject to transient HL including (i) de-epoxidation state (DPS) of xanthophyll cycle pigments, (ii) induction/relaxation of nonphotochemical quenching (parameterized as YNPQ), (iii) inactivation/repair of PSII and (iv) O_2_ consuming pathways (LDR/R_DARK_) for the three *Thalassiosira* diatom species examined here.

Diatoms exhibit varying responses to light to thrive across diverse environmental niches [40, 41, 101, 104–106]. Estuarine diatoms (e.g. *T*. *weissflogii*), exhibit a high capacity to rapidly initiate nonphotochemical quenching whereas oceanic diatoms (e.g. *T*. *oceanica*) have slower initiation of nonphotochemical quenching as light intensifies, with coastal diatoms (e.g. *T*. *pseudonana*), exhibiting an intermediate response [8, 107]. Our data confirmed these trends, despite similar rates of NPP across the three diatom representatives when grown under the same conditions of moderate, steady light (Table 1). Here, we add to the understanding of adaptive differences in photophysiological trade-offs employed by diatoms shifted to high light through (i) nonphotochemical quenching induction, (ii) reliance on energy consumption downstream of PSII and (iii) utilization of energetically expensive repair processes to counter damage to the photosynthetic machinery (Fig 8). Faster induction of nonphotochemical quenching was accompanied by lower susceptibility to PSII inactivation, while faster relaxation of nonphotochemical quenching corresponded with faster repair, across the three species. The faster nonphotochemical quenching relaxation and PSII repair for *T*. *pseudonana* was accompanied by greatest change in DPS capacity from Ig to HL. For *T*. *oceanica*, slower nonphotochemical quenching induction, and greatest susceptibility to PSII inactivation, was in turn accompanied by greater proportion of O_2_ evolved from PSII (GP_O2_) flowing to LDR, and less to R_DARK_. Thus, under transient high light exposure *T*. *weissflogii* adopts a strategy of rapid and sustained PSII downregulation, thereby requiring relatively little RCII inactivation/repair, or the need to induce O_2_ and electron consuming (LDR) pathways. In contrast, *T*. *oceanica* appears to not initiate protective mechanisms to alleviate excess excitation pressure on PSII, as evident by relatively little downregulation, inactivation and only moderate repair, but, instead, places greater reliance on LDR to dissipate excess excited energy downstream of PSII. Although these experiments were conducted under nutrient repletion, *T*. *oceanica* is evolved for low nutrient growth. Limiting reliance upon PSII repair thereby lowers the requirement for mineral nutrient investment into metabolically expensive systems for protein turnover [32, 108]. The response for coastal *T*. *pseudonana* is intermediate, with moderate downregulation and inactivation of PSII, but high repair of PSII and relaxation of nonphotochemical quenching, and some LDR. These trends show inherent trade-offs in how these different species deploy downregulation and repair of PSII, versus modulating subsequent re-consumption of oxygen and electrons (Fig 8).

In diatoms, activation of nonphotochemical quenching requires both the proton (H^+^) gradient across the thylakoid membrane (ΔpH) and xanthophyll cycling (XC), involving the de-epoxidation state (DPS) of diadinoxanthin (Dd, light harvesting pigment) to diatoxanthin (Dt, photo-protective pigment) [109]. Dt epoxidation to Dd in *T*. *pseudonana* was shown to be inhibited at HL due to the presence of a proton gradient, which maintains high concentrations of this photo-protective pigment. Dt epoxidation is also inhibited by complete darkness after HL exposure [85]. Such inhibition of Dt epoxidation allows diatoms to re-activate nonphotochemical quenching rapidly if needed, thus avoiding over-reliance on a single photo-protective mechanism [109]. Diatoms also benefit from rapid pigment conversion by Dt epoxidase during subsequent transition to low light that is evident through rapid relaxation/reversibility (within 5 min) of a component of nonphotochemical quenching [85, 110]. Such patterns were consistent with those we observed, with all species increasing Dt concentrations under HL [39], however this was not always consistent with a rapidly reversible nonphotochemical quenching. While *T*. *pseudonana* showed rapidly reversible nonphotochemical quenching, *T*. *weissflogii* appeared to sustain nonphotochemical quenching upon transition to low light, in parallel with its low capacity for PSII repair (k_REC_; Table 3). Sustained nonphotochemical quenching has been observed to have a linear relationship with Dt whereby at lower acclimated growth irradiances sustained nonphotochemical quenching at the initial dark fluorescence measure was around 5-fold lower than in high-light acclimated *T*. *gravida* [38]. However, recent studies have observed a deviation from this linearity [41], supporting the hypothesis that some portion of Dt is not directly related to nonphotochemical quenching and prevents full relaxation of nonphotochemical quenching [15]. While our study only obtained Dt concentrations at Ig and a brief (10 min) transient shift to HL, we cannot rule out the effect of Dt on sustained nonphotochemical quenching, and thus fluorescence signals retrieved. However, there was no significant difference in Dt among species at Ig (*p* = 0.323), therefore the trends observed appear robust. Interestingly, *T*. *oceanica* had slow initiation of nonphotochemical quenching under HL (Fig 1A) suggesting that a high content of Dt could be present but disconnected from RCII as was observed by Zhu & Green [10].

*T*. *oceanica* does not rapidly initiate nonphotochemical quenching to dissipate excess incident light energy in the antennae bed and, subsequently, suffers high excitation pressure on the RCIIs that split water and, potentially, higher excitation pressure through the subsequent electron carrier network. Increased ‘traffic’ of excitation energy was clear from the higher photo-inactivation rates (k_PI_) for *T*. *oceanica* compared to the other two species. Previous studies on diatoms have established a link between diatom cell size and susceptibility to photo-inactivation, whereby cell size is inversely proportional to photo-inactivation [73] and thus larger cells require lower PSII protein turnover [108]. This complements our data of higher PSII repair rates (k_REC_) for the smaller *T*. *pseudonana* than the larger *T*. *weissflogii*. Importantly, cell size may explain some photo-inactivation trends, but protein synthesis and regeneration, that alters in accordance with photosynthetic architecture also needs to be considered [32, 73]. It is technically difficult to discriminate between photoinactivation of PSII and sustained downregulation of PSII, but ecophysiologically [86] a sustained suppression of PSII activity imposes opportunity costs on subsequent productivity, whatever the mechanism.

PSII repair comes at a significant cost to the cell where the (re)synthesis of photosynthetic machinery comes at the expense of photosynthetic production [86]. Chloroplastic protein metabolism for PSII repair saturates at low light and continues during dark periods thus competing with growth for energy generated by photosynthesis [108, 111]. For *T*. *oceanica* with the highest k_PI_ (Table 3), alternative mechanisms may be employed to obtain additional metabolic energy at the expense of biosynthetic reductant. One source of energy could be PSII-MOX [50, 112] or PSI-Mehler [113] that consume O_2_ and generate a trans-membrane proton gradient to power ATP generation. While specific O_2_-consuming pathways were not distinguished in this study, there was evidence to support a higher reliance on energy sourced from O_2_-consuming pathways by *T*. *oceanica* evidenced by higher LDR at HL compared to all other species (S2 Table, Fig 5). The corresponding slower induction of nonphotochemical quenching exhibited by *T*. *oceanica* confirms previous studies showing a higher dependence on MOX processes. Importantly, such LDR pathways also act to consume excessive oxygen, which in the presence of high excitation pressure increases the chance of reactive oxygen species generation and further PSII—and indeed cellular—damage. Interestingly, *T*. *weissflogii* exhibited the highest dark respiration (S1 Table). A recent energetic coupling was found in diatoms between mitochondria and chloroplasts whereby ATP is supplied to the plastid by the mitochondria in the dark via upregulation of mitochondrial alternative oxidase (AOX) [60, 114, 115]. ATPase in the chloroplast hydrolyses this ATP to ADP which increases H^+^ concentration in the lumen that ultimately activates de-epoxidation of Dd to Dt [15]. Thus, in contrast to *T*. *oceanica* that is slower to initiate nonphotochemical quenching, our data would suggest *T*. *weissflogii* relies on “front loading”, or priming the photosynthetic apparatus, for rapid HL exposure at any time and in the absence of a light-driven proton motive force by keeping pH and Dt concentrations optimal for photo-protection.

Diatoms exhibit distinct alterations in photosynthetic architecture based on ecological niche, where oceanic diatoms (*T*. *oceanica*) have been found to have up to 10 PSII:PSI while coastal diatoms generally have 2 PSII:PSI [107]. These differences are primarily attributed to iron (Fe) availability, as the requirement for synthesis of PSII, Cyt b_6_f, and PSI are 3, 6, and 12 Fe atoms, respectively, but also provide insight into potential evolutionarily conserved species-specific photo-protective strategies amongst diatoms. Fe availability greatly influences growth rates of diatoms from various habitats whereby *T*. *pseudonana* and *T*. *weissflogii* growth rates were lowered by approximately 75% under Fe limitation while *T*. *oceanica* showed no significant change in growth rate [116] suggesting an evolutionary predisposition for the Fe-depleted open ocean. Different diatoms are equipped (genetically) to exploit many environments [2, 60, 117]. When light is stable and nutrients are limiting, typical of oceanic waters, diatoms appear to focus on upregulating light harvesting to produce more photochemical energy for cellular maintenance as nutrients are the limiting factor for division in these environments [101]. This pattern is consistent with the reliance of *T*. *oceanica* upon recycling electrons back to O_2_ under excess light. This cyclic flux of electrons trades biosynthetic reductant for ATP generation. If inorganic nutrients are limiting, the requirement for ATP for maintenance and nutrient uptake increases relative to the requirement for actual reductive biosynthesis. Conversely, for coastal/estuarine waters, where light is dynamic and nutrients plentiful, diatoms can afford to invest more energy in biosynthesis of macromolecules and division as well as energetically expensive photosynthetic machinery, such as PSI, that are more efficient trapping excitation energy than PSII [118]. Also, PSI photochemistry incurs a higher Fe requirement compared to ATP generation through MOX pathways [50]. The most studied MOX, plastid terminal oxidase (PTOX), was found to be a significant contributor to electron flow in marine *Synechococcus* [51] but absent for several coastal phytoplankton species compared to oceanic species [119, 120]. Based on our observations, we propose that *T*. *oceanica* cannot “afford” to synthesize new photosynthetic machinery and instead evolved strategies to allocate harvested light energy towards chemical energy for maintenance and growth while the slowly induced nonphotochemical quenching provides a fail-safe in the event of prolonged light stress.

## Conclusions

In summary, we have built on previous studies demonstrating differences in nonphotochemical quenching amongst diatom species, and strategies in dealing with transient high light exposure (e.g. [8]) to elucidate the trade-offs amongst varying energy dissipating strategies from ecologically distinct diatoms. We found that *T*. *weissflogii* and *T*. *pseudonana* exhibited capacity to rapidly initiate nonphotochemical quenching at lower light, which corresponded to lower light dependent respiration (LDR) at HL and lower k_PI_. *T*. *oceanica*, on the other hand, does not initiate nonphotochemical quenching as a rapid primary response mechanism to dissipate excess light energy and therefore had an accumulation of photochemical energy resulting in higher rates of LDR but also higher k_PI_. This supports the idea that photo-protective strategies are evolutionarily conserved based on ecological niche for diatoms. These diatoms possess similar core machinery to dissipate excess light energy but have balanced the mechanistic dissipation strategies employed to best suit their respective niche.

## Supporting information

S1 Fig Photophysiological assessment of seven diatoms. Conventional non-photochemical quenching (NPQ; see Eq 2) capacities of *Phaeodactylum tricornutum* (orange inverted triangles), *Chaetoceros muelleri* (yellow circles), *Ditylum brightwellii* (green diamonds), *Thalassiosira rotula* (black Xs), *Thalassiosira pseudonana* (black squares), *Thalassiosira weissflogii* (blue triangles), and *Thalassisosira oceanica* (red circles) with increasing light intensity. Error bars represent the standard error of the mean of at least n = 3 for independent biological replicates.
(TIF)

S1 Table Rates of oxygen production and consumption. (DOCX)

S2 Table MIMS analysis of oxygen pathways as a percentage of gross oxygen production (% of GP_O2_). (DOCX)

## References

[1] Tréguer P, Nelson DM, Van Bennekom AJ, Demaster DJ, Leynaert A, Quéguiner B. The silica balance in the world ocean: A reestimate. Science. 1995;268(5209):375–9. 10.1126/science.268.5209.375 17746543

[2] Armbrust EV. The life of diatoms in the world’s oceans. Nature. 2009;459(7244):185–92. 10.1038/nature08057 19444204

[3] Malviya S, Scalco E, Audic S, Vincent F, Veluchamy A, Poulain J, et al Insights into global diatom distribution and diversity in the world’s ocean. Proc Natl Acad Sci U S A. 2016;113(11):E1516–25. 10.1073/pnas.1509523113 26929361 PMC4801293

[4] Lavaud J, Goss R. The Peculiar Features of Non-Photochemical Fluorescence Quenching in Diatoms and Brown Algae. Non-Photochemical Quenching and Energy Dissipation in Plants, Algae and Cyanobacteria. 2014 421–443. 10.1007/978-94-017-9032-1_20

[5] Wilhelm C, Jungandreas A, Jakob T, Goss R. Light acclimation in diatoms: From phenomenology to mechanisms. Mar Genomics. 2014;16(1):5–15. 10.1016/j.margen.2013.12.003 24412570

[6] Behrenfeld MJ, Boss E, Siegel DA, Shea DM. Carbon-based ocean productivity and phytoplankton physiology from space. Global Biogeochem Cycles. 2005;19(1):1–14. 10.1029/2004GB002299

[7] Westberry T, Behrenfeld MJ, Siegel DA, Boss E. Carbon-based primary productivity modeling with vertically resolved photoacclimation. Global Biogeochem Cycles. 2008;22(2):1–18. 10.1029/2007GB003078

[8] Lavaud J, Strzepek RF, Kroth PG. Photoprotection capacity differs among diatoms: possible consequences on the spatial distribution of diatoms related to fluctuations in the underwater light Climate. Limnol Oceanogr. 2007;52(3):1188–94. 10.2307/4499689

[9] Dimier C, Corato F, Tramontano F, Brunet C. Photoprotection and xanthophyll-cycle activity in three marine diatoms. J Phycol. 2007;43(5):937–47. 10.1111/j.1529-8817.2007.00381.x

[10] Zhu SH, Green BR. Photoprotection in the diatom Thalassiosira pseudonana: Role of LI818-like proteins in response to high light stress. Biochim Biophys Acta—Bioenerg. 2010;1797(8):1449–57. 10.1016/j.bbabio.2010.04.003 20388491

[11] Goss R, Jakob T. Regulation and function of xanthophyll cycle-dependent photoprotection in algae. Photosynth Res. 2010;106(1–2):103–22. 10.1007/s11120-010-9536-x 20224940

[12] Lavaud J, Lepetit B. An explanation for the inter-species variability of the photoprotective non-photochemical chlorophyll fluorescence quenching in diatoms. Biochim Biophys Acta—Bioenerg. 2013;1827(3):294–302. 10.1016/j.bbabio.2012.11.012 23201475

[13] Campbell DA, Serôdio J. Photoinhibition of Photosystem II in Phytoplankton: Processes and Patterns In: Larkum A., Grossmann A., Raven J. (eds) Photosynthesis in Algae: Biochemical and Physiological Mechanisms. Advances in Photosynthesis and Respiration (Including Bioenergy and Related Processes). 2020 vol 45 Springer, Cham 10.1007/978-3-030-33397-3_13

[14] Waring J, Klenell M, Bechtold U, Underwood GJC, Baker NR. Light-induced responses of oxygen photoreduction, reactive oxygen species production and scavenging in two diatom species. J Phycol. 2010;46(6):1206–17. 10.1111/j.1529-8817.2010.00919.x

[15] Goss R, Lepetit B. Biodiversity of NPQ. J Plant Physiol. 2015;172:13–32. 10.1016/j.jplph.2014.03.004 24854581

[16] Klughammer C, Schreiber U. Complementary PS II quantum yields calculated from simple fluorescence parameters measured by PAM fluorometry and the Saturation Pulse method. PAM Appl Notes [Internet]. 2008;1(1):27–35. https://doi.org/citeulike-article-id:6352156

[17] Suggett DJ, Goyen S, Pettay DT, Szabó M, Warner ME, Evenhuis C, et al Functional diversity of photobiological traits within the genus Symbiodinium appears to be governed by the interaction of cell size with cladal designation. New Phytol. 2015;208(2):370–81. 10.1111/nph.13483 26017701

[18] Holt NE, Fleming GR, Niyogi KK. Toward an understanding of the mechanism of nonphotochemical quenching in green plants. Biochemistry. 2004;43(26):8281–9. 10.1021/bi0494020 15222740

[19] Lavaud J, Rousseau B, Etienne AL. General features of photoprotection by energy dissipation in planktonic diatoms (Bacillariophyceae). J Phycol. 2004;40(1):130–7. 10.1046/j.1529-8817.2004.03026.x

[20] Kramer DM, Avenson TJ, Edwards GE. Dynamic flexibility in the light reactions of photosynthesis governed by both electron and proton transfer reactions. Trends Plant Sci. 2004;9(7):349–57. 10.1016/j.tplants.2004.05.001 15231280

[21] Barnett A, Méléder V, Blommaert L, Lepetit B, Gaudin P, Vyverman W, et al Growth form defines physiological photoprotective capacity in intertidal benthic diatoms. ISME J. 2015;9(1):32–45. 10.1038/ismej.2014.105 25003964 PMC4274417

[22] Dong HP, Dong YL, Cui L, Balamurugan S, Gao J, Lu SH, et al High light stress triggers distinct proteomic responses in the marine diatom Thalassiosira pseudonana. BMC Genomics. 2016;17(1):1–14. 10.1186/s12864-016-3335-5 27919227 PMC5139114

[23] Lepetit B, Gélin G, Lepetit M, Sturm S, Vugrinec S, Rogato A, et al The diatom Phaeodactylum tricornutum adjusts nonphotochemical fluorescence quenching capacity in response to dynamic light via fine-tuned Lhcx and xanthophyll cycle pigment synthesis. New Phytol. 2017;214(1):205–18. 10.1111/nph.14337 27870063

[24] Taddei L, Chukhutsina VU, Lepetit B, Stella GR, Bassi R, van Amerongen H, et al Dynamic Changes between Two LHCX-Related Energy Quenching Sites Control Diatom Photoacclimation. Plant Physiol. 2018;177(3):953–65. 10.1104/pp.18.00448 29773581 PMC6053010

[25] Perkins R, Williamson C, Lavaud J, Mouget JL, Campbell DA. Time-dependent upregulation of electron transport with concomitant induction of regulated excitation dissipation in Haslea diatoms. Photosynth Res. 2018;137(3):377–88. 10.1007/s11120-018-0508-x 29663190 PMC6182385

[26] Lohr M, Wilhelm C. Algae displaying the diadinoxanthin cycle also possess the violaxanthin cycle. Proc Natl Acad Sci U S A. 1999;96(15):8784–9. 10.1073/pnas.96.15.8784 10411953 PMC17594

[27] Grouneva I, Jakob T, Wilhelm C, Goss R. Influence of ascorbate and pH on the activity of the diatom xanthophyll cycle-enzyme diadinoxanthin de-epoxidase. Physiol Plant. 2006;126(2):205–11. 10.1111/j.1399-3054.2006.00613.x

[28] Lepetit B, Goss R, Jakob T, Wilhelm C. Molecular dynamics of the diatom thylakoid membrane under different light conditions. Photosynth Res. 2012;111(1–2):245–57. 10.1007/s11120-011-9633-5 21327535

[29] Blommaert L, Huysman MJJ, Vyverman W, Lavaud J, Sabbe K. Contrasting NPQ dynamics and xanthophyll cycling in a motile and a non-motile intertidal benthic diatom. Limnol Oceanogr. 2017;62(4):1466–79. 10.1002/lno.10511

[30] Gao G, Shi Q, Xu Z, Xu J, Campbell DA, Wu H. Global warming interacts with ocean acidification to alter PSII function and protection in the diatom Thalassiosira weissflogii. Environ Exp Bot. 2018;147:95–103. 10.1016/j.envexpbot.2017.11.014

[31] Buck JM, Sherman J, Bártulos CR, Serif M, Halder M, Henkel J, et al Lhcx proteins provide photoprotection via thermal dissipation of absorbed light in the diatom Phaeodactylum tricornutum. Nat Commun. 2019;10(4167). 10.1038/s41467-019-12043-6 31519883 PMC6744471

[32] Campbell DA, Hossain Z, Cockshutt AM, Zhaxybayeva O, Wu H, Li G. Photosystem II protein clearance and FtsH function in the diatom Thalassiosira pseudonana. Photosynth Res. 2013;115(1):43–54. 10.1007/s11120-013-9809-2 23504483

[33] Giovagnetti V, Ruban A V. Detachment of the fucoxanthin chlorophyll a/c binding protein (FCP) antenna is not involved in the acclimative regulation of photoprotection in the pennate diatom Phaeodactylum tricornutum. Biochim Biophys Acta—Bioenerg. 2017;1858(3):218–30. 10.1016/j.bbabio.2016.12.005 27989819

[34] Kuzminov FI, Gorbunov MY. Energy dissipation pathways in Photosystem II of the diatom, Phaeodactylum tricornutum, under high-light conditions. Photosynth Res. 2016;127(2):219–35. 10.1007/s11120-015-0180-3 26220363

[35] Miloslavina Y, Grouneva I, Lambrev PH, Lepetit B, Goss R, Wilhelm C, et al Ultrafast fluorescence study on the location and mechanism of non-photochemical quenching in diatoms. Biochim Biophys Acta—Bioenerg. 2009;1787(10):1189–97. 10.1016/j.bbabio.2009.05.012 19486881

[36] Lepetit B, Volke D, Gilbert M, Wilhelm C, Goss R. Evidence for the existence of one antenna-associated, lipid-dissolved and two protein-bound pools of diadinoxanthin cycle pigments in diatoms. Plant Physiol. 2010;154(4):1905–20. 10.1104/pp.110.166454 20935178 PMC2996015

[37] Lepetit B, Sturm S, Rogato A, Gruber A, Sachse M, Falciatore A, et al High light acclimation in the secondary plastids containing diatom phaeodactylum tricornutum is triggered by the redox state of the plastoquinone pool. Plant Physiol. 2013;161(2):853–65. 10.1104/pp.112.207811 23209128 PMC3561024

[38] Lacour T, Larivière J, Ferland J, Bruyant F, Lavaud J, Babin M. The role of sustained photoprotective non-photochemical quenching in low temperature and high light acclimation in the bloom-forming arctic diatom Thalassiosira gravida. Front Mar Sci. 2018;5:1–16. 10.3389/fmars.2018.0035429552559

[39] Lacour T, Babin M, Lavaud J. Diversity in Xanthophyll Cycle Pigments Content and Related Nonphotochemical Quenching (NPQ) Among Microalgae: Implications for Growth Strategy and Ecology. J Phycol. 2020;56(2):245–63. 10.1111/jpy.12944 31674660

[40] Derks AK, Bruce D. Rapid regulation of excitation energy in two pennate diatoms from contrasting light climates. Photosynth Res. 2018;138(2):149–65. 10.1007/s11120-018-0558-0 30008155 PMC6208626

[41] Croteau D, Guérin S, Bruyant F, Ferland J, Campbell DA, Babin M, et al Contrasting nonphotochemical quenching patterns under high light and darkness aligns with light niche occupancy in Arctic diatoms. Limnol Oceanogr. 2020;1–15. 10.1002/lno.11587

[42] Lavaud J, Six C, Campbell DA. Photosystem II repair in marine diatoms with contrasting photophysiologies. Photosynth Res. 2016;127(2):189–99. 10.1007/s11120-015-0172-3 26156125

[43] Cardol P, Forti G, Finazzi G. Regulation of electron transport in microalgae. Biochim Biophys Acta—Bioenerg. 2011;1807(8):912–8. 10.1016/j.bbabio.2010.12.004 21167125

[44] Alric J, Johnson X. Alternative electron transport pathways in photosynthesis: a confluence of regulation. Curr Opin Plant Biol. 2017;37:78–86. 10.1016/j.pbi.2017.03.014 28426976

[45] Hughes DJ, Campbell DA, Doblin MA, Kromkamp JC, Lawrenz E, Moore CM, et al Roadmaps and Detours: Active Chlorophyll- a Assessments of Primary Productivity Across Marine and Freshwater Systems. Environ Sci Technol. 2018;52(21):12039–54. 10.1021/acs.est.8b03488 30247887

[46] Lavaud J, Van Gorkom HJ, Etienne AL. Photosystem II electron transfer cycle and chlororespiration in planktonic diatoms. Photosynth Res. 2002;74(1):51–9. 10.1007/s11120-015-0172-3 16228544

[47] Onno Feikema W, Marosvölgyi MA, Lavaud J, van Gorkom HJ. Cyclic electron transfer in photosystem II in the marine diatom Phaeodactylum tricornutum. Biochim Biophys Acta—Bioenerg. 2006;1757(7):829–34. https://10.1016/j.bbabio.2006.06.003 16857159 10.1016/j.bbabio.2006.06.003

[48] Wagner H, Jakob T, Lavaud J, Wilhelm C. Photosystem II cycle activity and alternative electron transport in the diatom Phaeodactylum tricornutum under dynamic light conditions and nitrogen limitation. Photosynth Res. 2016;128(2):151–61. https://10.1007/s11120-015-0209-7 26650230 10.1007/s11120-015-0209-7

[49] Schreiber U. Pulse-Amplitude-Modulation (PAM) Fluorometry and Saturation Pulse Method: An Overview In: Papageorgiou G.C., Govindjee (eds) Chlorophyll a Fluorescence. Advances in Photosynthesis and Respiration. 2004 vol 19 Springer, Dordrecht 10.1007/978-1-4020-3218-9_11

[50] Behrenfeld MJ, Halsey KH, Milligan AJ. Evolved physiological responses of phytoplankton to their integrated growth environment. Philos Trans R Soc B Biol Sci. 2008;363(1504):2687–703. 10.1098/rstb.2008.0019 18487129 PMC2606763

[51] Bailey S, Melis A, Mackey KRM, Cardol P, Finazzi G, van Dijken G, et al Alternative photosynthetic electron flow to oxygen in marine Synechococcus. Biochim Biophys Acta—Bioenerg. 2008;1777(3):269–76. 10.1016/j.bbabio.2008.01.002 18241667

[52] Nawrocki WJ, Tourasse NJ, Taly A, Rappaport F, Wollman F-A. The Plastid Terminal Oxidase: Its Elusive Function Points to Multiple Contributions to Plastid Physiology. Annu Rev Plant Biol. 2015;66(1):49–74. 10.1146/annurev-arplant-043014-114744 25580838

[53] Asada K. The water-water cylce in chloroplasts: scavenging of active oxygens and dissipation of excess photons. Annu Rev Plant Physiol Plant Mol Biol. 1999;50(1):601–39. 10.1146/annurev.arplant.50.1.601 15012221

[54] Weger HG, Herzig R, Falkowski PG, Turpin DH. Respiratory losses in the light in a marine diatom: measuremetns by short-term mass spectrometry. Limnol Oceanogr. 1989;34(7):1153–61.

[55] Allahverdiyeva Y, Suorsa M, Tikkanen M, Aro EM. Photoprotection of photosystems in fluctuating light intensities. J Exp Bot. 2015;66(9):2427–36. 10.1093/jxb/eru463 25468932

[56] Kana TM, Darkangelo C, Hunt MD, Oldham JB, Bennett GE, Cornwell JC. Membrane lnlet Mass Spectrometer for Rapid Environmental Water Samples. Response. 1994;66(23):4166–70. http://pubs.acs.org/doi/abs/10.1021/ac00095a009

[57] Fisher NL, Halsey KH. Mechanisms that increase the growth efficiency of diatoms in low light. Photosynth Res. 2016;129(2):183–97. 10.1007/s11120-016-0282-6 27312336

[58] Kromkamp JC, Peene J. Oxygen consumption in the light by unicellular algae. Science Access. 2001;3(1).

[59] Claquin P, Kromkamp JC, Martin-Jezequel V. Relationship between photosynthetic metabolism and cell cycle in a synchronized culture of the marine alga Cylindrotheca fusiformis (Bacillariophyceae). Eur J Phycol. 2004;39(1):33–41. 10.1080/0967026032000157165

[60] Murik O, Tirichine L, Prihoda J, Thomas Y, Araújo WL, Allen AE, et al Downregulation of mitochondrial alternative oxidase affects chloroplast function, redox status and stress response in a marine diatom. New Phytol. 2019;221(3):1303–16. 10.1111/nph.15479 30216452

[61] Raven JA, Kübler JE, Beardall J. Put out the light, and then put out the light. J Mar Biol Assoc United Kingdom. 2000;80(1):1–25. https://doi.org/10/.1017/S0025315499001526

[62] Giordano M, Norici A, Hell R. Sulfur and phytoplankton: Acquisition, metabolism and impact on the environment. New Phytol. 2005;166(2):371–82. 10.1111/j.1469-8137.2005.01335.x 15819903

[63] Guillard RL. Culture of phytoplankton for feeding marine invertebrates In: Culture of Marine Invertebrate Animals. 1975 p. 29–60.

[64] Laws EA, Bannister TT. Nutrient- and light-limited growth of Thalassiosira fluviatilis in continuous culture with implications for phytoplankton growth in the ocean. *Limnology and Oceanography*, 25(3), 457–473. 10.4319/lo.2004.49.6.2316

[65] Hughes DJ, Varkey D, Doblin MA, Ingleton T, Mcinnes A, Ralph PJ, et al Impact of nitrogen availability upon the electron requirement for carbon fixation in Australian coastal phytoplankton communities. Limnol Oceanogr. 2018;63(5):1891–910. 10.1002/lno.10814

[66] Kolber ZS, Prasil O, Falkowski PG. Measurements of variable chlorophyll fluorescence using fast repetition rate techniques: defining methodology and experimental protocols. Biochim Biophys Acta—Bioenerg. 1998;1367:88–106. http://publication/uuid/C591C675-7666-49B5-AB3C-16E2C3FCFD57 9784616 10.1016/s0005-2728(98)00135-2

[67] Bilger W, Björkman O. Role of the xanthophyll cycle in photoprotection elucidated by measurements of light-induced absorbance changes, fluorescence and photosynthesis in leaves of Hedera canariensis. Photosynth Res. 1990;25(3):173–85. 10.1007/BF00033159 24420348

[68] Serôdio J, Cruz S, Vieira S, Brotas V. Non-photochemical quenching of chlorophyll fluorescence and operation of the xanthophyll cycle in estuarine microphytobenthos. J Exp Mar Bio Ecol. 2005;326(2):157–69. https://10.1016/j.jembe.2005.05.011

[69] Genty B, Harbinson J. Regulation of Light Utilization for Photosynthetic Electron Transport. Photosynth Environ. 2006;67–99. https://10.1007/0-306-48135-9_3

[70] Oxborough K, Baker NR. Resolving chlorophyll a fluorescence images of photosynthetic efficiency into photochemical and non-photochemical components—Calculation of qP and Fv’/Fm’ without measuring Fo’. Photosynth Res. 1997;54(2):135–42. 10.1023/A:1005936823310

[71] Sun J, Liu D. Geometric models for calculating cell biovolume and surface area for phytoplankton. J Plankton Res. 2003;25(11),1331–1346. 10.1093/plankt/fbg096

[72] Ritchie RJ. Consistent sets of spectrophotometric chlorophyll equations for acetone, methanol and ethanol solvents. Photosynth Res. 2006;89(1):27–41. 10.1007/s11120-006-9065-9 16763878

[73] Key T, McCarthy A, Campbell DA, Six C, Roy S, Finkel ZV. Cell size trade-offs govern light exploitation strategies in marine phytoplankton. Environ Microbiol. 2010;12(1):95–104. 10.1111/j.1462-2920.2009.02046.x 19735282

[74] Wu H, Roy S, Alami M, Green BR, Campbell DA. Photosystem II photoinactivation, repair, and protection in marine centric diatoms. Plant Physiol. 2012;160(1):464–76. 10.1104/pp.112.203067 22829321 PMC3440219

[75] Li G, Woroch AD, Donaher NA, Cockshutt AM, Campbell DA. A Hard Day’s Night: Diatoms Continue Recycling Photosystem II in the Dark. Front Mar Sci. 2016;3:1–10. 10.3389/fmars.2016.00218

[76] Kok B. On the inhibition of photosynthesis by intense light. Biochim. Biophys. Acta. 1956;21:234–244. 10.2514/3.29809 13363902

[77] Oliver RL, Whittington J, Lorenz Z, Webster IT. Erratum: The influence of vertical mixing on the photoinhibition of variable chlorophyll-a fluorescence and its inclusion in a model of phytoplankton photosynthesis. J Plankton Res. 2013;35(4):927 10.1093/plankt/25.9.1107

[78] Campbell DA, Tyystjärvi E. Parameterization of photosystem II photoinactivation and repair. Biochim Biophys Acta—Bioenerg [Internet]. 2012;1817(1):258–65. 10.1016/j.bbabio.2011.04.010 21565161

[79] Elzhov TV, Mullen KM, Spiess AN, Bolker B. minpack. lm: R interface to the Levenberg-Marquardt nonlinear least-squares algorithm found in MINPACK, plus support for bounds. R package version 1.2–1. https://CRAN.R-project.org/package=minpack.lm

[80] R Core Team. R: A language and environment for statistical computing. R Foundation for Statistical Computing, Vienna, Austria https://www.R-project.org/

[81] RStudio Team. RStudio: Integrated Development for R. RStudio. PBC Boston, MA http://www.rstudio.com/

[82] Wickham H. ggplot2: elegant graphics for data analysis. Springer-Verlag New York 2016 6 8 http://www.springer.com/gp/book/9783319242750

[83] Heukelem L, Thomas CS. Computer-assisted high-performance liquid chromatography method development with applications to the isolation and analysis of phytoplankton pigments. *J*. *Chromat*. *A*. 2001;910(1),31–49. https://doi.org/10.1016/S0378-4347(00)00603-4 123 11263574 10.1016/s0378-4347(00)00603-4

[84] Grouneva I, Jakob T, Wilhelm C, Goss R. The regulation of xanthophyll cycle activity and of non-photochemical fluorescence quenching by two alternative electron flows in the diatoms Phaeodactylum tricornutum and Cyclotella meneghiniana. Biochim Biophys Acta—Bioenerg. 2009;1787(7):929–38. 10.1016/j.bbabio.2009.02.004 19232316

[85] Goss R, Ann Pinto E, Wilhelm C, Richter M. The importance of a highly active and ΔpH-regulated diatoxanthin epoxidase for the regulation of the PS II antenna function in diadinoxanthin cycle containing algae. J Plant Physiol. 2006;163(10):1008–21. 10.1016/j.jplph.2005.09.008 16971213

[86] Raven JA. The cost of photoinhibition. Physiol Plant. 2011;142(1):87–104. 10.1111/j.1399-3054.2011.01465.x 21382037

[87] Stransky H, Hager A. The carotenoid pattern and the occurrence of the light-induced xanthophyll cycle in various classes of algae. IV. Cyanophyceae and Rhodophyceae. Arch Mikrobiol. 1970;72(1),84–96. 4988022

[88] Horton P, Ruban A V. Regulation of Photosystem II. Photosynth Res. 1992;34(3):375–85. https://10.1007/BF00029812 24408833 10.1007/BF00029812

[89] Suggett DJ, MacIntyre HL, Kana TM, Geider RJ. Comparing electron transport with gas exchange: Parameterising exchange rates between alternative photosynthetic currencies for eukaryotic phytoplankton. Aquat Microb Ecol. 2009;56(2–3):147–62. 10.3354/ame01303

[90] Halsey KH, Milligan AJ, Behrenfeld MJ. Physiological optimization underlies growth rate-independent chlorophyll-specific gross and net primary production. Photosynth Res. 2010;103(2):125–37. 10.1007/s11120-009-9526-z 20066494

[91] Halsey KH, O’Malley RT, Graff JR, Milligan AJ, Behrenfeld MJ. A common partitioning strategy for photosynthetic products in evolutionarily distinct phytoplankton species. New Phytol. 2013;198(4):1030–8. 10.1111/nph.12209 23452244

[92] Mckew BA, Davey P, Finch SJ, Hopkins J, Lefebvre SC, Metodiev M V., et al The trade-off between the light-harvesting and photoprotective functions of fucoxanthin-chlorophyll proteins dominates light acclimation in Emiliania huxleyi (clone CCMP 1516). New Phytol. 2013;200(1):74–85. 10.1111/nph.12373 23790241

[93] Du N, Gholami P, Kline DI, DuPont CL, Dickson AG, Mendola D, et al Simultaneous quantum yield measurements of carbon uptake and oxygen evolution in microalgal cultures. PLoS One. 2018;13(6):1–21. 10.1371/journal.pone.0199125 29920568 PMC6008153

[94] Brading P, Warner ME, Davey P, Smith DJ, Achterberg EP, Suggett DJ. Differential effects of ocean acidification on growth and photosynthesis among phylotypes of Symbiodinium (Dinophyceae). Limnol Oceanogr. 2011;56(3):927–38. 10.4319/lo.2011.56.3.0927

[95] Ferrón S, del Valle DA, Bjöorkman KM, Quay PD, Church MJ, Karl DM. Application of membrane inlet mass spectrometry to measure aquatic gross primary production by the 18O in vitro method. Limnology and Oceanography: Methods. 2016;14(9),610–622. 10.1002/lom3.10116

[96] Ananda MMA, Weerahandi S. Two-Way ANOVA with unequal cell frequencies and unequal variances. Stat Sin. 1997;7(3):631–46.

[97] Anderson M, Gorley R, Clarke KP. For PRIMER: guide to software and statistical methods PRIMER-E. 2008 Plymouth, UK.

[98] Walter B, Peters J, van Beusekom JEE, John MA St.. Interactive effects of temperature and light during deep convection: a case study on growth and condition of the diatom Thalassiosira weissflogii. ICES J Mar Sci. 2015;72(6):2061–71. 10.1093/icesjms/fsu218

[99] Lomas MW. Nitrate reductase and urease enzyme activity in the marine diatom Thalassiosira weissflogii (Bacillariophyceae): Interactions among nitrogen substrates. Mar Biol. 2004;144(1):37–44. 10.1007/s00227-003-1181-x

[100] Bucciarelli E, Ridame C, Sunda WG, Dimier-Hugueney C, Cheize M, Belviso S. Increased intracellular concentrations of DMSP and DMSO in iron-limited oceanic phytoplankton Thalassiosira oceanica and Trichodesmium erythraeum. Limnol Oceanogr. 2013;58(5):1667–79. 10.4319/lo.2013.58.5.1667

[101] Suggett DJ, Moore CM, Hickman AE, Geider RJ. Interpretation of fast repetition rate (FRR) fluorescence: Signatures of phytoplankton community structure versus physiological state. Mar Ecol Prog Ser. 2009;376:1–19. 10.3354/meps07830

[102] Suggett DJ, Oxborough K, Baker NR, Macintyre HL, Kana TM, Geider RJ. Fast repetition rate and pulse amplitude modulation chlorophyll a fluorescence measurements for assessment of photosynthetic electron transport in marine phytoplankton. Eur J Phycol. 2003;38(4):371–84. https://10.1080/09670260310001612655

[103] Waring J, Klenell M, Bechtold U, Underwood GJC, Baker NR. Light-induced responses of oxygen photoreduction, reactive oxygen species production and scavenging in two diatom species. J Phycol. 2010;46(6):1206–17. https://10.1111/j.1529-8817.2010.00919.x

[104] Larkum AWD. Light-Harvesting Systems in Algae. 2003 277–304. 10.1007/978-94-007-1038-2_13

[105] Litchman E, Klausmeier CA, Bossard P. Phytoplankton nutrient competition under dynamic light regimes. Limnol Oceanogr. 2004;49(4part2):1457–62. 10.4319/lo.2004.49.4_part_2.1457

[106] Edwards KF, Thomas MK, Klausmeier CA, Litchman E. Light and growth in marine phytoplankton: Allometric, taxonomic, and environmental variation. Limnol Oceanogr. 2015;60(2):540–52. 10.1002/lno.10033

[107] Strzepek RF, Harrison PJ. Photosynthetic architecture differs in coastal and oceanic diatoms. Nature. 2004;431(7009):689 10.1038/nature02954 15470428

[108] Li G, Brown CM, Jeans JA, Donaher NA, Mccarthy A, Campbell DA. The nitrogen costs of photosynthesis in a diatom under current and future pCO2. New Phytol. 2015;205(2):533–43. 10.1111/nph.13037 25256155

[109] Lavaud J, Rousseau B, Van Gorkom HJ, Etienne AL. Influence of the diadinoxanthin pool size on photoprotection in the marine planktonic diatom Phaeodactylum tricornutum. Plant Physiol. 2002;129(3):1398–406. 10.1104/pp.002014 12114593 PMC166533

[110] Milligan AJ, Aparicio UA, Behrenfeld MJ. Fluorescence and nonphotochemical quenching responses to simulated vertical mixing in the marine diatom Thalassiosira weissflogii. Mar Ecol Prog Ser. 2012;448:67–78. 10.3354/meps09544

[111] Li G, Woroch AD, Donaher NA, Cockshutt AM, Campbell DA. A Hard Day’s Night: Diatoms Continue Recycling Photosystem II in the Dark. Frontiers in Marine Science. 2016;3:1–10. 10.3389/fmars.2016.00218

[112] Behrenfeld MJ, Milligan AJ. Photophysiological Expressions of Iron Stress in Phytoplankton. Ann Rev Mar Sci. 2013;5:217–46. 10.1146/annurev-marine-121211-172356 22881354

[113] Miyake C, Suzuki Y, Yamamoto H, Amako K, Makino A. O2-enhanced induction of photosynthesis in rice leaves: the Mehler-ascorbate peroxidase (MAP) pathway drives cyclic electron flow within PSII and cyclic electron flow around PSI. Soil Sci Plant Nutr. 2012;58(6):718–27. 10.1080/00380768.2012.736078

[114] Prihoda J, Tanaka A, De Paula WBM, Allen JF, Tirichine L, Bowler C. Chloroplast-mitochondria cross-talk in diatoms. J Exp Bot. 2012;63(4):1543–57. 10.1093/jxb/err441 22268145

[115] Bailleul B, Berne N, Murik O, Petroutsos D, Prihoda J, Tanaka A, et al Energetic coupling between plastids and mitochondria drives CO 2 assimilation in diatoms. Nature. 2015;524(7565):366–9. 10.1038/nature14599 26168400

[116] Maldonado MT, Price NM. Influence of N substrate on Fe requirements of marine centric diatoms. Mar Ecol Prog Ser. 1996;141(1–3):161–72. 10.3354/meps141161

[117] Lommer M, Specht M, Roy AS, Kraemer L, Andreson R, Gutowska MA, et al Genome and low-iron response of an oceanic diatom adapted to chronic iron limitation. Genome Biol. 2012;13(7):R66 10.1186/gb-2012-13-7-r66 22835381 PMC3491386

[118] Joly D, Carpentier R. Regulation of energy dissipation in photosystem I by the redox state of the plastoquinone pool. Biochemistry. 2007;46(18):5534–41. 10.1021/bi602627d 17432831

[119] Mackey KRM, Paytan A, Grossman AR, Bailey S. A photosynthetic strategy for coping in a high-light, low-nutrient environment. Limnol Oceanogr. 2008;53(3):900–13. 10.4319/lo.2008.53.3.0900

[120] Rusch DB, Martiny AC, Dupont CL, Halpern AL, Venter JC. Characterization of Prochlorococcus clades from iron-depleted oceanic regions. Proc Natl Acad Sci U S A. 2010;107(37):16184–9. 10.1073/pnas.1009513107 20733077 PMC2941326

