# Supplementary figures and images for: Divergence of photosynthetic strategies amongst marine diatoms
Source: PLoS One. 2020 Dec 28;15(12):e0244252. doi: 10.1371/journal.pone.0244252 (PMC7769462; doi:10.1371/journal.pone.0244252)

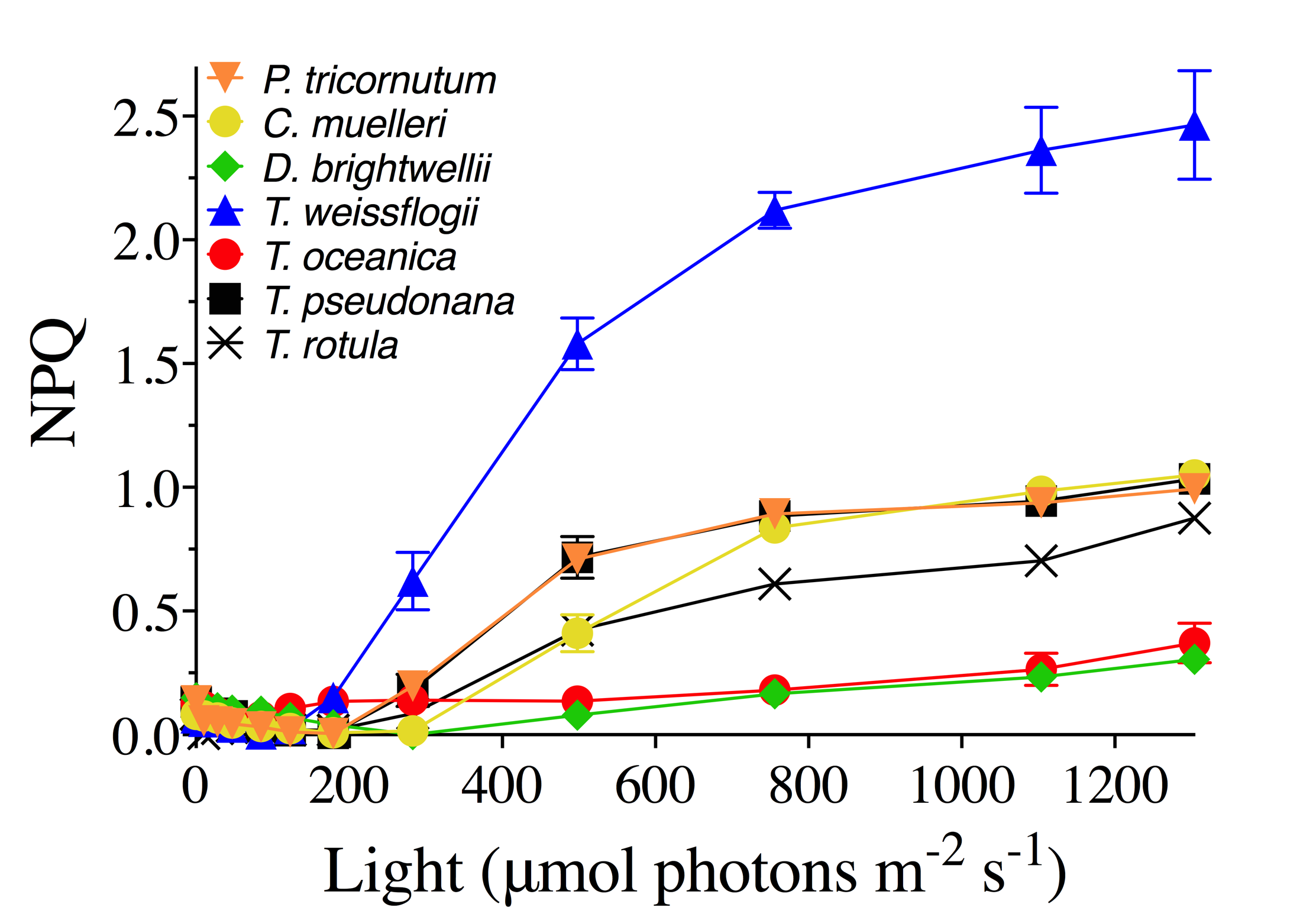

Supplement: S1 Fig — Conventional non-photochemical quenching (NPQ; see Eq 2) capacities of Phaeodactylum tricornutum (orange inverted triangles), Chaetoceros muelleri (yellow circles), Ditylum brightwellii (green diamonds), Thalassiosira rotula (black Xs), Thalassiosira pseudonana (black squares), Thalassiosira weissflogii (blue triangles), and Thalassisosira oceanica (red circles) with increasing light intensity. Error bars represent the standard error of the mean of at least n = 3 for independent biological replicates. (TIF) [file pone.0244252.s001.tif]
