# Supplementary material for: Divergence of photosynthetic strategies amongst marine diatoms
Source: PLoS One. 2020 Dec 28;15(12):e0244252. doi: 10.1371/journal.pone.0244252 (PMC7769462; doi:10.1371/journal.pone.0244252)
Supplement: S1 Table — (DOCX) [file pone.0244252.s002.docx]

**S1 Table. Rates of oxygen production and consumption.**

| **Species** | **Light** | **pmol cell^-1^ h^-1^** | | | | | | |
| --- | --- | --- | --- | --- | --- | --- | --- | --- |
|  |  | **R_DARK_** | **LDR** | | | **Net_O2_** | **GP_O2_** | |
| ***T. weissflogii*** | Ig | 0.16^b^  (0.03) | 0.06 (0.03) | | | 0.32 (0.12) | 0.54 (0.13) | |
|  | HL |  | 0.06 (0.02) | | | 0.64 (0.15) | 0.86 (0.18) | |
| ***T. oceanica*** | Ig | 0.02^a^  (0.00) | 0.02 (0.00) | | | 0.14 (0.04) | 0.18 (0.04) | |
|  | HL |  | 0.04 (0.01) | | | 0.14 (0.04) | 0.20 (0.05) | |
| ***T. pseudonana*** | Ig | 0.02^a^  (0.01) | 0.01 (0.00) | | | 0.08 (0.01) | 0.11 (0.02) | |
|  | HL |  | 0.02 (0.01) | | | 0.09 (0.02) | 0.13 (0.02) | |
| ANOVA  (1-way) | p-value | **<0.05** | n/a | | | n/a | n/a | |
| ANOVA  (2-way) | p-value | Species | | >0.05 | **<0.05**^*^ | | | **<0.05**^*^ |
|  |  | Light | | >0.05 | >0.05 | | | >0.05 |
|  |  | Species*Light | | >0.05 | >0.05 | | | >0.05 |

MIMS analysis of gross oxygen production (GP_O2_), light dependent respiration (LDR), dark respiration (R_DARK_), and net oxygen production (Net_O2_) for *T. weissflogii*, *T. oceanica*, and *T. pseudonana* under 20 min exposure to HL (1200 µmol photons m^-2^ s^-1^). Data averaged from 3 independent replicates. Values in parentheses are SE of the mean. A 2-way ANOVA test comparing species and light treatment with oxygen measurements (GP_O2_, Net_O2_, LDR) and a 1-way ANOVA for R_DARK_ are presented with significant p-values (< 0.05) in bold. Superscripted asterisks (*) or letter identifies the Bonferroni post-hoc analysis for significance between species groups.

^*^ *T. weissflogii* – *T. oceanica*/*T. pseudonana*
