# Supplementary material for: Divergence of photosynthetic strategies amongst marine diatoms
Source: PLoS One. 2020 Dec 28;15(12):e0244252. doi: 10.1371/journal.pone.0244252 (PMC7769462; doi:10.1371/journal.pone.0244252)
Supplement: S2 Table — (DOCX) [file pone.0244252.s003.docx]

**S2 Table. MIMS analysis of oxygen pathways as a percentage of gross oxygen production (% of GP_O2_).**

| **Species** | | **Light** | **% of GP_O2_** | | | | | |
| --- | --- | --- | --- | --- | --- | --- | --- | --- |
|  |  |  | **R_DARK_** | | | **LDR** | **Net_O2_** | |
| ***T. weissflogii*** | | Ig | 21.67 (3.33) | | | 11.83 (4.35) | 66.50 (1.02) | |
|  |  | HL | 19.33 (2.17) | | | 8.07 (2.81) | 72.60 (4.98) | |
| ***T. oceanica*** | | Ig | 9.38 (2.45) | | | 10.98 (0.22) | 79.64 (2.66) | |
|  |  | HL | 8.46 (2.34) | | | 17.72 (2.50) | 73.82 (0.17) | |
| ***T. pseudonana*** | | Ig | 20.02 (4.67) | | | 9.98 (3.35) | 69.99 (4.57) | |
|  |  | HL | 16.58 (4.03) | | | 15.42 (6.15) | 68.01 (2.47) | |
| ANOVA  (2-way) | Species | | | **<0.05**^*^ | >0.05 | | | >0.05 |
|  | Light | | | >0.05 | >0.05 | | | >0.05 |
|  | Species*Light | | | >0.05 | >0.05 | | | >0.05 |

Fractions (as a %) include light dependent respiration (LDR), dark respiration (R_DARK_), and net oxygen production (Net_O2_) for *T. weissflogii*, *T. oceanica*, and *T. pseudonana* under 20 min incubation at Ig (85 µmol photons m^-2^ s^-1^) and HL (1200 µmol photons m^-2^ s^-1^). Data averaged from 2 or 3 independent replicates. Values in parentheses are SE of the mean. A 2-way ANOVA comparing species and light treatment with oxygen measurements (R_DARK_, Net_O2_, LDR) are presented with significant p-values (< 0.05) in bold. Superscripted asterisks (*) identifies the Fisher’s Tukey post-hoc analysis for significance between species groups.

^*^ *T. oceanica* – *T. weissflogii*/*T. pseudonana*
